# Recombinant collagen for the repair of skin wounds and photo-aging damage

**DOI:** 10.1093/rb/rbae108

**Published:** 2024-09-02

**Authors:** Taishan Liu, Jiayun Hao, Huan Lei, Yanru Chen, Lin Liu, Liping Jia, Juan Gu, Huaping Kang, Jingjing Shi, Jing He, Yangbin Song, Yuqi Tang, Daidi Fan

**Affiliations:** Shaanxi Key Laboratory of Degradable Biomedical Materials, School of Chemical Engineering, Northwest University, Xi’an 710069, China; Shaanxi R&D Center of Biomaterials and Fermentation Engineering, School of Chemical Engineering, Northwest University, Xi’an 710069, China; Biotech & Biomed Research Institute, Northwest University, Xi’an 710069, China; Shaanxi Key Laboratory of Degradable Biomedical Materials, School of Chemical Engineering, Northwest University, Xi’an 710069, China; Shaanxi R&D Center of Biomaterials and Fermentation Engineering, School of Chemical Engineering, Northwest University, Xi’an 710069, China; Biotech & Biomed Research Institute, Northwest University, Xi’an 710069, China; Shaanxi Key Laboratory of Degradable Biomedical Materials, School of Chemical Engineering, Northwest University, Xi’an 710069, China; Shaanxi R&D Center of Biomaterials and Fermentation Engineering, School of Chemical Engineering, Northwest University, Xi’an 710069, China; Biotech & Biomed Research Institute, Northwest University, Xi’an 710069, China; Shaanxi Key Laboratory of Degradable Biomedical Materials, School of Chemical Engineering, Northwest University, Xi’an 710069, China; Shaanxi R&D Center of Biomaterials and Fermentation Engineering, School of Chemical Engineering, Northwest University, Xi’an 710069, China; Biotech & Biomed Research Institute, Northwest University, Xi’an 710069, China; Xi’an Giant Biotechnology Co. Ltd., Xi’an 710100, China; Xi’an Giant Biotechnology Co. Ltd., Xi’an 710100, China; Shaanxi Giant Biotechnology Co. Ltd., Xi'an 710076, China; Shaanxi Giant Biotechnology Co. Ltd., Xi'an 710076, China; Shaanxi Giant Biotechnology Co. Ltd., Xi'an 710076, China; Xi’an Giant Biotechnology Co. Ltd., Xi’an 710100, China; Shaanxi Giant Biotechnology Co. Ltd., Xi'an 710076, China; Shaanxi Giant Biotechnology Co. Ltd., Xi'an 710076, China; Shaanxi Key Laboratory of Degradable Biomedical Materials, School of Chemical Engineering, Northwest University, Xi’an 710069, China; Shaanxi R&D Center of Biomaterials and Fermentation Engineering, School of Chemical Engineering, Northwest University, Xi’an 710069, China; Biotech & Biomed Research Institute, Northwest University, Xi’an 710069, China

**Keywords:** recombinant collagen, wound repair, skin photo-aging repair, cell proliferation

## Abstract

The skin, being the body’s primary defense mechanism, is susceptible to various injuries such as epidermal wounds, natural aging, and ultraviolet-induced damage. As a result, there is growing interest in researching skin repair methods. Traditional animal-derived collagen, widely available on the market, poses risks due to its immunogenicity and potential for viral contamination. In contrast, recombinant collagen sourced from human genes offers a safer alternative. To investigate the potential of human recombinant collagen in skin repair, our research team applied two types, type I human collagen (Col I) and CF-1552(I), to two different skin injury models: a wound-healing model and a photo-aging model. Our findings indicate that both Col I and CF-1552(I) effectively enhance wound healing and repair skin damaged by ultraviolet exposure. Notably, CF-1552(I) showed effects comparable to Col I in promoting cell proliferation in the wound-healing model and increasing malondialdehyde content in the photo-aging model, suggesting that CF-1552(I) may offer greater potential for skin repair compared to the larger Col I molecule.

## Graphical Abstract

**Figure:**
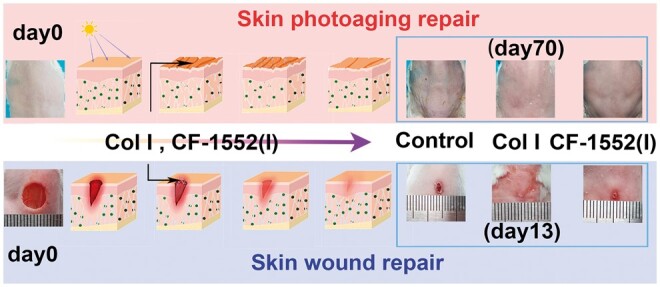

## Introduction

The skin, the largest organ in the human body, functions as the main interface with the external environment. It plays a crucial role in regulating body temperature, sensing pain and stress, defending against pathogens and protecting against ultraviolet (UV) radiation [1, 2]. As the body’s primary defense mechanism, the skin is constantly exposed to daily damage from trauma, bacterial infections, burns, natural aging and UVB-induced aging [3]. This ongoing and intricate process of skin repair underscores its significant value [4, 5].

Collagen is extensively utilized in skin repair research due to its significant biological properties as an extracellular matrix (ECM) component in the human body. It plays a crucial role in supporting the normal physiological functions of cells, tissues and organs, and it positively affects trauma repair, skin regeneration and photo-aging recovery [6–8]. Currently, animal-derived collagen, mainly bovine collagen, is predominantly used in biomedical, pharmaceutical and tissue-engineered medical applications. However, this reliance on animal collagen raises several safety concerns, including risks of pathogenicity, virus transmission, diseases and allergic reactions [9, 10]. Specifically, transmissible spongiform encephalopathy, such as bovine spongiform encephalopathy, poses a significant restriction on the use of bovine collagen from infected regions [11]. Additionally, traditional treatments for animal collagen, like heat treatment, often fail to preserve its structural and functional integrity, further complicating its use [12, 13]. Consequently, these issues highlight the limitations of traditional collagen extraction methods, impeding progress in collagen research and development [14–16].

These phenomena highlight that animal-derived collagen still presents biosafety concerns, underscoring the need for a safe and reliable alternative that is both biocompatible and biologically effective [17]. Our previous research led to the development of recombinant type I human collagen (Col I), which addresses biosafety issues by eliminating the risk of animal-borne infectious diseases and demonstrates enhanced bio-effectiveness with excellent biocompatibility [18–20]. Concurrently, our team employed DNA recombinant technology to create recombinant type I humanized collagen (CF-1552(I)) by expressing the functional amino acid sequence fragment of human collagen. This humanized collagen not only maintains the characteristics of conventional collagen but also boasts improved water solubility, reduced immune rejection and superior biological compatibility [21, 22]. CF-1552(I) is derived from the human type I collagen gene chain but differs from Col I, which encompasses the entire gene sequence of the type I collagen α1(I) chain and closely mirrors natural type I collagen. In contrast, CF-1552(I) is based on a 15-amino-acid fragment of the type I collagen gene, which, after codon optimization and splicing recombination, results in a new recombinant humanized collagen sequence [23, 24]. The smaller molecular weight of CF-1552(I) compared to Col I enhances its water solubility and enables rapid degradation into a single helix structure suitable for injury site applications. Furthermore, CF-1552(I) has optimized the -(GXY) sequence of the α(I) peptide chain and removed amino acid residues like tryptophan that could cause immune reactions, further minimizing allogeneic rejection. The unique repetitive structure of CF-1552(I), due to its amino acid sequence, allows chemically active amino acids to bind precisely to their target sites, significantly enhancing collagen flexibility.

This study explores the efficacy of Col I and CF-1552 in promoting wound healing and repairing UV-induced skin photo-aging. It also examines the mechanisms by which Col I and CF-1552(I)—a repetitive 15-amino-acid sequence in type I collagen—facilitate these processes. The unique repeatability of CF-1552(I) may enhance skin repair by leading to a higher content of effective gene fragments post-hydrolysis. Additionally, CF-1552(I) exhibits excellent water solubility, making it easier to degrade into peptides that penetrate the skin more effectively and act within cells. The study also focuses on how CF-1552(I) may improve the activity of certain intracellular genes.

## Materials and methods

### Materials

Recombinant Col I (Presented by Shaanxi Giant Biotechnology Co. Ltd., Mw: 110 kDa). Recombinant CF-1552(I) (Presented by Shaanxi Giant Biotechnology Co. Ltd., [HPLC] ≥ 99%, Mw: 97 kDa). The amino acid sequence of Col I and CF-1552 is shown in Table 1. DMEM was purchased from HyClone (Logan, UT, USA). Methylthiazolyldiphenyl tetrazolium bromide (MTT) was purchased from Beyotime Biotechnology (Shanghai, China). Bovine Collagen (BC) (Beijing, China). Trypsin and penicillin–streptomycin (pen–strep) were purchased from Solarbio Science & Technology Co., Ltd (Beijing, China). Fetal bovine serum was purchased from Biological Industries (Cromwell, CT, USA). Human skin fibroblasts (HSF) and human immortalized keratinocytes (HACAT) were obtained from Fu Heng Biology (Shanghai, China).

**Table 1.** The amino acid sequence of COLI and CF1552

Collagen types	Sequence
Col I	QLSYGYDEKSTGGISVPGPMGPSGPRGLPGPPGAPGPQGFQGPPGEPGEPGASGPMGPRGPPGPPGKNGDDGEAGKPGRPGERGPPGPQGARGLPGTAGLPGMKGHRGFSGLDGAKGDAGPAGPKGEPGSPGENGAPGQMGPRGLPGERGRPGAPGPAGARGNDGATGAAGPPGPTGPAGPPGFPGAVGAKGEAGPQGPRGSEGPQGVRGEPGPPGPAGAAGPAGNPGADGQPGAKGANGAPGIAGAPGFPGARGPSGPQGPGGPPGPKGNSGEPGAPGSKGDTGAKGEPGPVGVQGPPGPAGEEGKRGARGEPGPTGLPGPPGERGGPGSRGFPGADGVAGPKGPAGERGSPGPAGPKGSPGEAGRPGEAGLPGAKGLTGSPGSPGPDGKTGPPGPAGQDGRPGPPGPPGARGQAGVMGFPGPKGAAGEPGKAGERGVPGPPGAVGPAGKDGEAGAQGPPGPAGPAGERGEQGPAGSPGFQGLPGPAGPPGNDGAKGDAGAPGAPGSQPGEAGKPGEQGVPGDLGAPGPSGARGERGFPGERGVQGPPGPAGPRGANGAGAPGLQGMPGERGAAGLPGPKGDRGDAGPKGADGSPGKDGVRGLTGPIGPPGPAGAPGDKGESGPSGPAGPTGARGAPGDRGEPGPPGPAGFAGPPGADGQPGAKGEPGDAGAKGDAGPPGPAGPAGPPGPIGNVGAPGAKGARGSAGPPGATGFPGAAGRVGPPGPSGNAGPPGPPGPAGKEGGKGPRGETGPAGRPGEVGPPGPPGPAGEKGSPGADGPAGAPGTPGPQGIAGQRGVVGLPGQRGERGFPGLPGPSGEPGKQGPSGASGERGPPGPMGPPGLAGPPGESGREGAPGAEGSPGRDGSPGAKGDRGETGPAGPPGAPGAPGAPGPVGPAGKSGDRGETGPAGPAGPVGPVGARGPAGPQGPRGDKGETGEQGDRGIKGHRGFSGLQGPPGPPGSPGEQGPSGASGPAGPRGPPGSAGAPGKDGLNGLPGPIGPPGPRGRTGDAGPVGPPGPPGPPGPPGPPSAGFDFSFLPQPPQEKAHDGGRYYRA
CF-1552(I)	MDPVVLQRRDWENPGVTQLNRLAAHPPFASDPM(GAPGAPGSQGAPGLQ) n GAMGSS, *n*=52

**Table 2.** Gene primers used in qPCR

Gene	Primer (5′–3′)	Primer direction
Col α(I)	5′-ACATGCCGTGACCTCAAGAT-3′	Forward
	5′-ATGTCCATTCCGAATTCCTG-3′	Reverse
TGFβ-1	5′-ATGGAGAGAGGACTGCGGAT-3′	Forward
	5′-TAGTGTTCCCCACTGGTCCC-3′	Reverse
VIM	5′-TCCGCACATTCGAGCAAAGA-3′	Forward
	5′-TGATTCAAGTCTCAGCGGGC-3′	Reverse

### *In vitro* analysis of cell proliferation

To assess cell proliferation using an MTT assay, skin keratinocytes and fibroblasts, which are essential for wound healing, were analyzed. HACAT and HSF cell lines were seeded into 96-well plates at a density of 5 × 10³ cells per well. After allowing the cells to adhere, the original culture medium was replaced, and the wells were rinsed with PBS. The control group was given a serum-free medium, while the experimental group received a serum-free medium containing the samples. Following a 48-h incubation, 50 μl of MTT solution (5 mg/ml) was added to each well. After 2 h, the MTT solution was removed, and 150 μl of DMSO was added to each well. Absorbance at 490 nm was then measured using a Bio-Tek microplate reader. Each concentration was tested in quintuplicate for analysis.

### *In vitro* study of cell migration

To investigate how collagen affects the migration of HACAT and HSF cells, an *in vitro* wound-healing model was set up. HACAT and HSF cells (1 × 10^6^ cells/well) were seeded into 6-well plates and cultured until a confluent monolayer formed. A wound was created by scratching the monolayer with a sterile 200 μl pipette tip, followed by rinsing with PBS to remove cell debris. The samples were then treated with collagen-containing medium (1 mg/ml) for further incubation. Images of the scratches were captured using a microscope, and the healing process was analyzed with ImageJ (×64) at 0 and 24 h.

### Real-time quantitative polymerase chain reaction

HACAT and HSF cells were cultured in a collagen-containing medium (1 mg/ml) to study its effects on cell differentiation, with the medium being replaced every 2 days for a total of four changes. After 1 week, cells were collected into separate centrifuge tubes for RNA extraction. They were lysed with Trizol reagent and incubated at room temperature for 8 min. The lysate was transferred to a new 2 ml microtube, incubated at 25°C for 10 min, mixed with 200 µl of chloroform, and incubated again at 25°C for 10 min. Following this, the mixture was centrifuged at 12 000 rpm for 20 min at 4°C. The supernatant was transferred to a new 1.5 ml microtube, mixed with isopropanol, and incubated at 25°C for 10 min. After centrifugation at 12 000 rpm for 10 min at 4°C, the supernatant was discarded, and the pellet was washed with 75% ethanol and centrifuged. The final pellet was dissolved in enzyme-free water. Complementary DNA (cDNA) synthesis was carried out using the RevertAid First Strand cDNA Synthesis Kit (Thermo Fisher Scientific, USA), and quantitative reverse transcriptase-polymerase chain reaction (RT-qPCR) was performed using a Bio-Rad system (USA). β-actin gene RNA expression served as a reference to normalize the RNA expression levels of other genes. The specific primers were shown in Table 2.

### Circular dichroism spectrum of collagen

To ensure proper calibration of the instrument, including zero calibration and wavelength accuracy, dilute the collagen samples to the appropriate concentration in a solution. First, measure the CD signal of the buffer alone, then measure the CD signal of the sample in the range of approximately 190–250 nm.

### Preparation of HACAT and HSF cell photo-aging model

Skin photo-aging primarily affects skin keratin-forming cells, so experiments were carried out using HACAT and HSF cells. These cells were cultured in dishes until they reached approximately 90% confluence, then seeded into 96-well plates at a density of 5000 cells per well. The wells were divided into control and UVB model groups. After 24 h of incubation, the culture medium was removed, the wells were washed with PBS, and each well was filled with 100 μl of PBS. The control wells, which did not require UVB irradiation, were covered with tin foil, while the UVB model wells were exposed to UVB irradiation for 4 h at 15 mJ/cm^2^ using a UVB lamp.

### *In vitro* effect of collagen on the proliferation of photo-aging cells

The same modeling procedure described in section “Circular dichroism spectrum of collagen” was applied using a collagen concentration of 1 mg/ml and UVB-type UV lamps with an irradiation intensity of 15 mJ/cm^2^. After 24 h of incubation, 20 μl of MTT solution (5 mg/ml in sterile PBS) was added, followed by the addition of 150 μl of DMSO after 4 h. The cells were then shaken for 10 min on a shaker. The absorbance at 490 nm was measured using an enzyme marker, and the survival rate for each group was calculated.

(1) $$\mathrm{Survival}\;\mathrm{rate}\;\left(\%\right)=\left(\frac{A_{\mathrm{model}\;\mathrm{group}}}{A_{\mathrm{control}\;\mathrm{group}}\;}\right)\times 100\%$$

### *In vitro* effect of collagen on the level of superoxide dismutase in photo-aged cells

HACAT and HSF cells were cultured in dishes until reaching approximately 90% confluence. The cells were then transferred to 6-well plates at a density of 1 × 10^6^ cells per well, grouped and treated according to the method described in section “Circular dichroism spectrum of collagen”. To disrupt the cells and release intracellular components, they were fragmented and centrifuged at 2000–3000 rpm for about 20 min. The supernatant was carefully collected, and the human superoxide dismutase (SOD) enzyme-linked immunoassay (ELISA) kit was used to test the sample. The procedure was followed according to the standard protocol, the absorbance at 450 nm was measured, and the SOD value was determined by comparing the results to a standard curve.

### *In vitro* effect of collagen on the levels of reactive oxygen species in photo-aged cells

HACAT and HSF cells were cultured in a dish until they reached approximately 90% confluence. They were then transferred to a 6-well plate at a density of 1 × 10^6^ cells per well, grouped, modeled, and treated according to the protocol outlined in section “Circular dichroism spectrum of collagen” for 24 h. After removing the culture medium, 1 ml of DCFH-DA (10 μmol/l), diluted 1:1000 in serum-free culture medium, was added. The cells were incubated at 37°C with 5% CO_2_ for 20 min, with the solution being gently mixed every 5 min to ensure thorough contact between the probe and the cells. The cells were then washed three times with serum-free culture medium followed by three washes with PBS. Finally, 1 ml of PBS was added, and the level of reactive oxygen species (ROS) damage was assessed using fluorescence microscopy for imaging.

### Wound-healing rate

All animal experiments were reviewed and approved by the Northwest University Laboratory Animal Management and Ethics Committee (approval number NWU-AWC-20220904M), ensuring compliance with legal and institutional animal ethics guidelines. Sixty female Kunming mice, each weighing 20 ± 2 g, were used to investigate the effects of collagen on wound healing. The mice were housed in laboratory facilities under a 12 h light/12 h dark cycle for 7 days to acclimate, with free access to food pellets and drinking water throughout the experiment. After shaving the dorsal hair of the mice, they were randomly divided into six groups of 10 mice each. Mice were anesthetized with an injectable 3% pentobarbital sodium solution at a dose of 1.0 ml/kg. A circular wound with an 8-mm diameter was created on the dorsal region, and a collagen solution (1 mg/ml) was applied to the wound using a pipette gun and evenly distributed with a cotton swab once daily. On days 0, 4, 8, and 13, optical images of the wounds were captured using a digital camera from five mice in each group to monitor healing progress, and wound areas were measured accurately using Image J software.

### Histological examinations and Masson staining

Skin samples from three mice were collected at 4, 8 and 13 days postoperatively, fixed in 10% formaldehyde for over 24 h, embedded in paraffin wax and sectioned into 5-μm slices. These slices were then subjected to hematoxylin and eosin (H&E) and Masson staining, and collagen density was analyzed using ImageJ.

### Immunofluorescence staining

The skin tissue sample, fixed with formaldehyde, was embedded in paraffin wax, cut and mounted on a glass slide. Immunofluorescence staining was conducted using α-smooth muscle actin (α-SMA; Servicebio, GB111364), Col I (Servicebio, GB11022-3), and collagen III (Col III; Servicebio, GB111269). The fluorescence intensity was then observed and analyzed using ImageJ.

### Animal model for photo-aging skin repair

Forty female Kunming mice (weighing 20 ± 2 g) were used to investigate the efficacy of collagen in repairing skin photo-aging. The mice were acclimatized for 7 days in a laboratory with a 12 h light/12 h dark cycle, having free access to food and water. Their back hair was shaved (3 cm × 3 cm), and they were then randomly assigned to four groups of 10 mice each. UVA and UVB lamps were positioned 15 cm from the mice’s skin to irradiate the exposed areas. The minimum erythema dose (MED) was measured using a UV energy detector, with UVA set at 1.71 J/cm^2^ and UVB at 0.39 J/cm^2^ for a 30-min irradiation. To create a UV-induced photo-aging model, the mice underwent two stages of UV irradiation: in the first phase, they received 1 MED every other day for the initial 5 weeks, and in the second phase, the dose was increased to 2 MED every other day for the following 5 weeks. The total UVA dose amounted to 92.34 J/cm^2^, and the total UVB dose was 21.06 J/cm^2^ over the 10-week period. Mice in the treatment group had collagen solution applied to their backs daily, those in the normal-blank group received no UV irradiation or treatment, mice in the untreated-collagen group were treated with collagen but not irradiated, and those in the model group were irradiated without treatment. If any mice developed ulcers or blisters, irradiation was paused 1–2 times until the symptoms resolved, after which the experiment resumed.

### Effect of collagen on malondialdehyde levels in the skin of photo-damaged mice

At the end of the 10th week, skin tissue was harvested from the backs of the mice following the procedures outlined in “Immunofluorescence staining,” and then cut and weighed. After adding an appropriate amount of PBS, the specimens were homogenized thoroughly. The homogenate was centrifuged at 2000–3000 rpm for approximately 20 min, and the supernatant was carefully collected. Using the human malondialdehyde (MDA) ELISA kit, the sample was processed according to the standard protocol. The absorbance at 450 nm was measured with an enzyme marker, and the MDA value of the sample was determined by comparing this measurement with the standard curve.

### Effect of collagen on hydroxyproline levels in the skin of photo-damaged mice

At the end of the 10th week following the grouping, modeling, and treatment methods described in “Immunofluorescence staining,” skin tissue from the back of the mice was collected, cut, and weighed. Approximately 0.2 g of the sample was placed in a glass tube, cut into small pieces to facilitate digestion, and the cap was left slightly loose. To the tube, 2 ml of extract was added, and the mixture was digested by boiling or in an oven at 110°C for 2–6 h, or until no large clumps remained. After digestion, the mixture was cooled, and the pH was adjusted to 6–8 using 10 mol/l NaOH (about 1 ml), ensuring not to over-acidify or over-base. Distilled water was added to bring the total volume to 4 ml. The mixture was then centrifuged at 16 000 rpm for 20 min at 25°C. If impurities remained after centrifugation, they were removed by filtration, and the supernatant was collected for measurement. Black material that might appear during digestion is likely carbonized and does not affect the experiment. The absorbance of the enzyme standard was measured at 560 nm and used to calculate the HYP content using a standard curve.

### Histological examinations and Masson staining

The mice were grouped, modeled, and treated as described in “Immunofluorescence staining.” At the end of the 10th week, skin tissue samples were collected from their backs and fixed in 10% formaldehyde for over 24 h. These samples were then embedded in paraffin wax, sectioned into 5-μm slices, and stained with H&E and Masson. Collagen density was assessed using ImageJ.

### Immunofluorescence staining

Skin tissue samples were fixed with formaldehyde, embedded in paraffin, and then sectioned and mounted on slides. To detect type I procollagen and MMP-1, mouse anti-human procollagen I and rabbit anti-human MMP-1 antibodies (FITC, 1:2000 dilution) were used. The fluorescence intensity was observed and analyzed using ImageJ.

### Statistical analyses

Data were expressed as mean ± SD and analyzed using SPSS (IBM SPSS Statistics 19) software. All experiments were repeated at least three times, and significance analysis included one-sided or bilateral *t*-tests. (Significance was as follows: compared to the normal group: *P <* *0.05; **P <* *0.01; ***P <* *0.001, compared to the control group: ^#^*P *<* *0.05; ^##^*P *<* *0.01; ^##^*P *<* *0.001.)

## Results and discussion

### *In vitro* Col I and CF-1552(I) promotes cell proliferation, migration and intracellular gene differentiation

To explore how collagen influences the intricate process of wound healing, we conducted *in vitro* experiments to assess the effects of Col I and CF-1552(I) on cell proliferation, migration, and intracellular gene differentiation [25–27].

#### Cell proliferation

**Figure 1.**
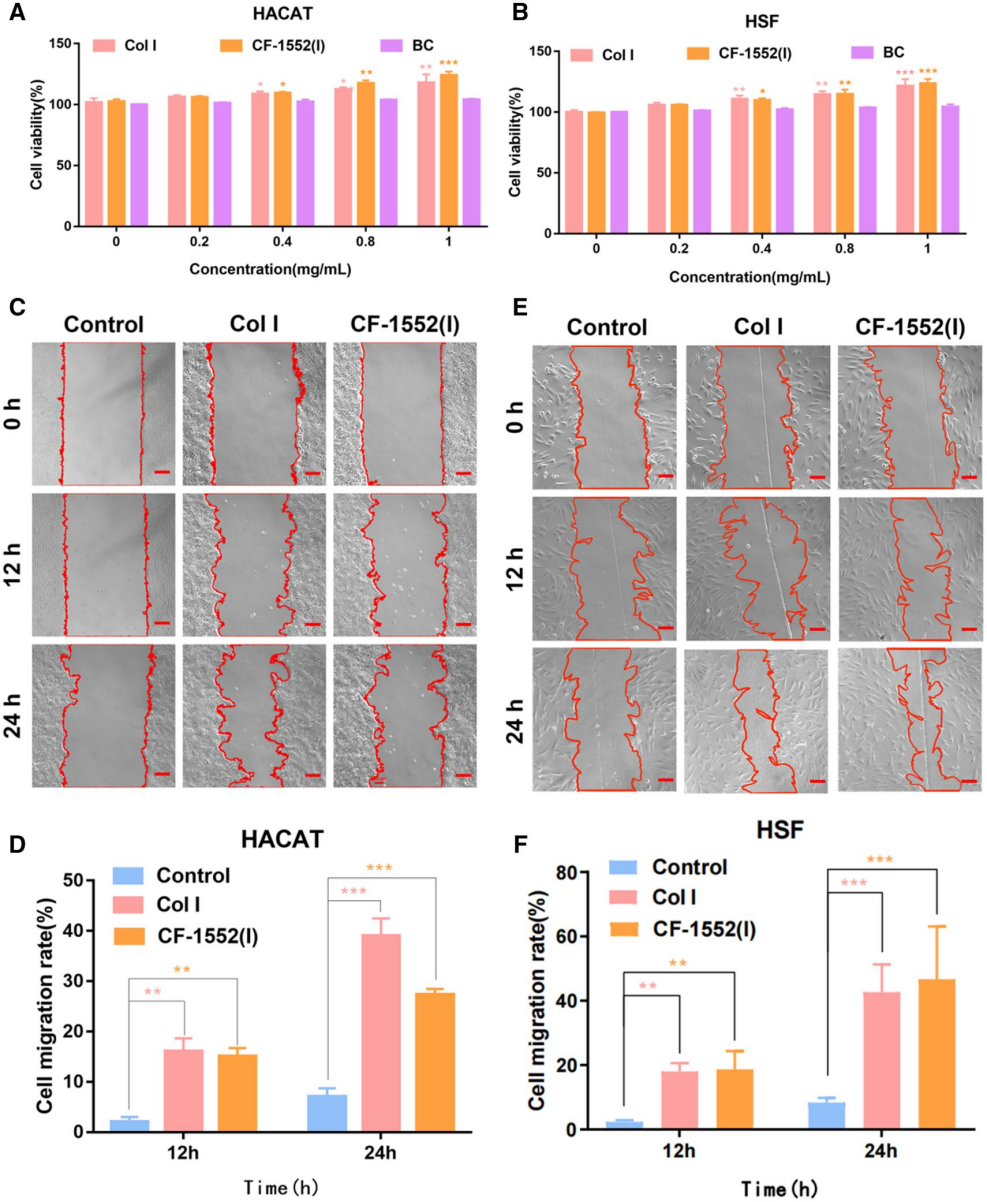
(**A**) Cell viability (HACAT) with collagen (*P < 0.05, **P < 0.01, *n* = 3) specify 100% cell viability for the control group using normal media; (**B**) cell viability (HSF) with collagen (**P < 0.01, *n* = 3) specify 100% cell viability for the control group using normal media; (**C**) microscope images of *in vitro* scratches for the various experimental groups of HACAT cells (scale bar: 50 μm); (**D**) quantified *in vitro* scratch closure results for the various experimental groups of HACAT cells (**P < 0.01, ***P < 0.001, *n* = 3); (**E**) microscope images of *in vitro* scratches for the various experimental groups of HSF cells (scale bar: 50 μm) and (**F**) quantified *in vitro* scratch closure results for the various experimental groups of HSF cells (**P < 0.01, ***P < 0.001, *n* = 3).

Skin wound healing is a complex physiological process, with re-epithelialization being a critical step that involves the migration and proliferation of keratinocytes and skin fibers to cover the exfoliated dermal surface [28]. Therefore, cells were cultured using collagen to observe cell proliferation. HACAT and HSF were treated with serum-free cultures containing different concentrations of Col I, CF-1552(I) and BC collagen (0, 0.2, 0.4, 0.8 and 1 mg/ml) for 48 h to assess cell viability. Figure 1A and B illustrates that cell proliferation rates increased with higher collagen concentrations. Specifically, HACAT and HSF cells treated with 1 mg/ml CF-1552(I) showed proliferation rates of 132.22% and 125.37%, respectively, compared to the control group (0 mg/ml), while cells treated with 1 mg/ml Col I had proliferation rates of 127.35% and 123.22%, respectively. In the early stages of wound repair, collagen promotes extensive proliferation of immortalized epidermal cells and fibroblasts. Subsequently, fibroblasts transform into myofibroblasts, aiding in wound contraction [29, 30]. The effectiveness of Col I and CF-1552(I) in enhancing skin healing at the cellular level was partially confirmed by their notable pro-proliferative properties. However, collagen BC from the bovine Achilles tendon did not exhibit significant effects on cell proliferation throughout the process, likely due to its insolubility in the neutral culture medium. Consequently, BC was not used in subsequent experiments for control.

#### In vitro wound closure

To study the effect of collagen on cell migration during the complex wound-healing process, we cultured cells in a collagen-containing medium and observed their migration, particularly in scratched areas [31, 32]. After treating HACAT cells with medium containing 1 mg/ml Col I and CF-1552(I) for 24 h, the cell migration rates were 37.5% (*P* < 0.001) and 29.5% (*P* < 0.001), respectively, as shown in Figure 1C and D. Similarly, treatment of HSF cells with the same medium for 24 h resulted in cell migration rates of 42.5% (*P* < 0.001) and 45.5% (*P* < 0.001), as depicted in Figure 1F and G. These results indicate that both Col I and CF-1552(I) effectively promote cell migration and accelerate cellular scratch healing, with no significant difference observed between the two treatments in enhancing HSF cell migration.

#### Intracellular gene differentiation

**Figure 2.**
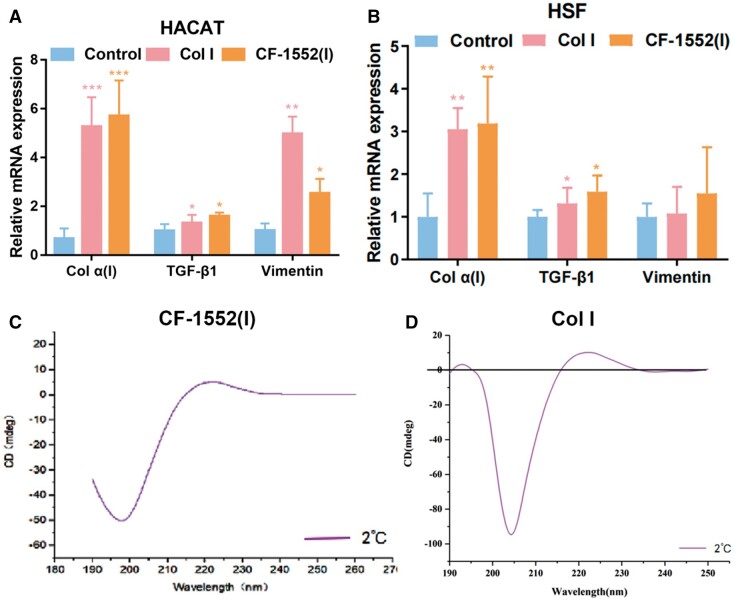
(**A**) Differentiation of promoted genes Col α(I), TGF-β1 and vimentin in cells (HACAT) treated with different collagen (*P <* *0.05, **P <* *0.01, ***P <* *0.001, *n* = 3); (**B**) differentiation of promoted genes Col I α(I), TGF-β1 and VIM in cells (HSF) treated with different collagen (*P <* *0.05, **P <* *0.01, *n* = 3); (**C**) circular dichroism of the CF-1552(I) materials and (**D**) circular dichroism of the Col I materials.

Collagen type I (Col α(I)), a crucial component of the ECM, plays a significant role in wound healing and tissue strength [33]. The fibroblast proliferation essential for this process relies on Transforming growth factor-β1 (TGF-β1） [34] and the introduction of fibroblasts enhances the synthesis and deposition of Col α(I), thereby increasing the tensile strength of the wound [35–37]. Additionally, myofibroblast contraction drives the wound-healing process. Genes and proteins influencing wound healing and differentiation are associated with vimentin (VIM) [38, 39]. To assess the impact of collagen on the promotion of intracellular Col α(I), TGF-β1, and VIM differentiation, RT-qPCR was employed. Cultures with collagen at 1 mg/ml concentration stimulated mRNA levels linked to HACAT and HSF differentiation. Figure 2A and B illustrates that inHACAT and HSF cells cultured with CF-1552(I), the expression of Col α(I) and TGF-β1 increased significantly by 5.9-fold (*P* < 0.001) and 1.67-fold (*P* < 0.05), respectively. Additionally, waveform protein expression rose by 2.7-fold (*P* < 0.05) compared to the control group. When comparing the effects of Col I and CF-1552(I) on gene differentiation, Col I treatment resulted in a 4.9-fold increase in waveform protein expression in HACAT cells, which was notably higher than the 2.7-fold increase observed with CF-1552(I) (*P* < 0.01). During the later stages of wound healing, the expression of Col α(I) and TGF-β1 proteins was significantly enhanced. Both Col I and CF-1552(I) promoted the differentiation of TGF-β1, leading to increased synthesis and deposition of Col α(I). However, there was no significant difference between Col I and CF-1552(I) in their ability to enhance TGF-β1 expression. VIM, which supports cytoskeletal stability, was also upregulated by both Col I and CF-1552(I), indicating their role in promoting cellular stability.

#### Triple helix structure of Col I and CF-1552(I) materials

In general, natural collagen is a typical triple-stranded superhelical structure assembled from three left α-helices twisted together, and its CD is usually characterized by displaying a significant negative absorption peak near 198 nm and a smaller positive absorption peak near 220 nm, and these two absorption peaks demonstrate the triple-helical structure of Col I [40–42] (Figure 2D). However, during the denaturation process of collagen, the absorption peak at 220 nm will decrease as the degree of denaturation deepens, while the absorption peak at 198 nm tends to be significant. As shown in Figure 2C, CF-1552(I) protein has a very obvious negative absorption peak at 198 nm, but a small and broad positive absorption peak at 220 nm, which may be due to the existence of an irregular coil structure in the secondary structure of the protein [43]. The spectral features of CF-1552(I) are similar to those of denatured collagen [44], which suggests that the CF-1552(I) protein is collagen that has not formed a three-stranded helical structure.

### Effect of *in vitro* Col I and CF-1552(I) on cellular-level photo-aging repair

UVB and, to a lesser extent, UVA irradiation cause damage to human skin, which leads to sun-induced skin diseases. UVB exposure initiates photo-oxidation reactions that disrupt antioxidant defenses and elevate cellular ROS levels. The extent of ROS-induced damage is moderated by antioxidants like SOD, which helps maintain physiological balance [45, 46]. Therefore, we investigated the effects of Col I and CF-1552(I) on intracellular ROS and SOD levels under UVB irradiation by *in vitro* experiments.

#### Col I and CF-1552(I) repair the cytotoxicity of UVB irradiation in HACAT and HSF cells

**Figure 3.**
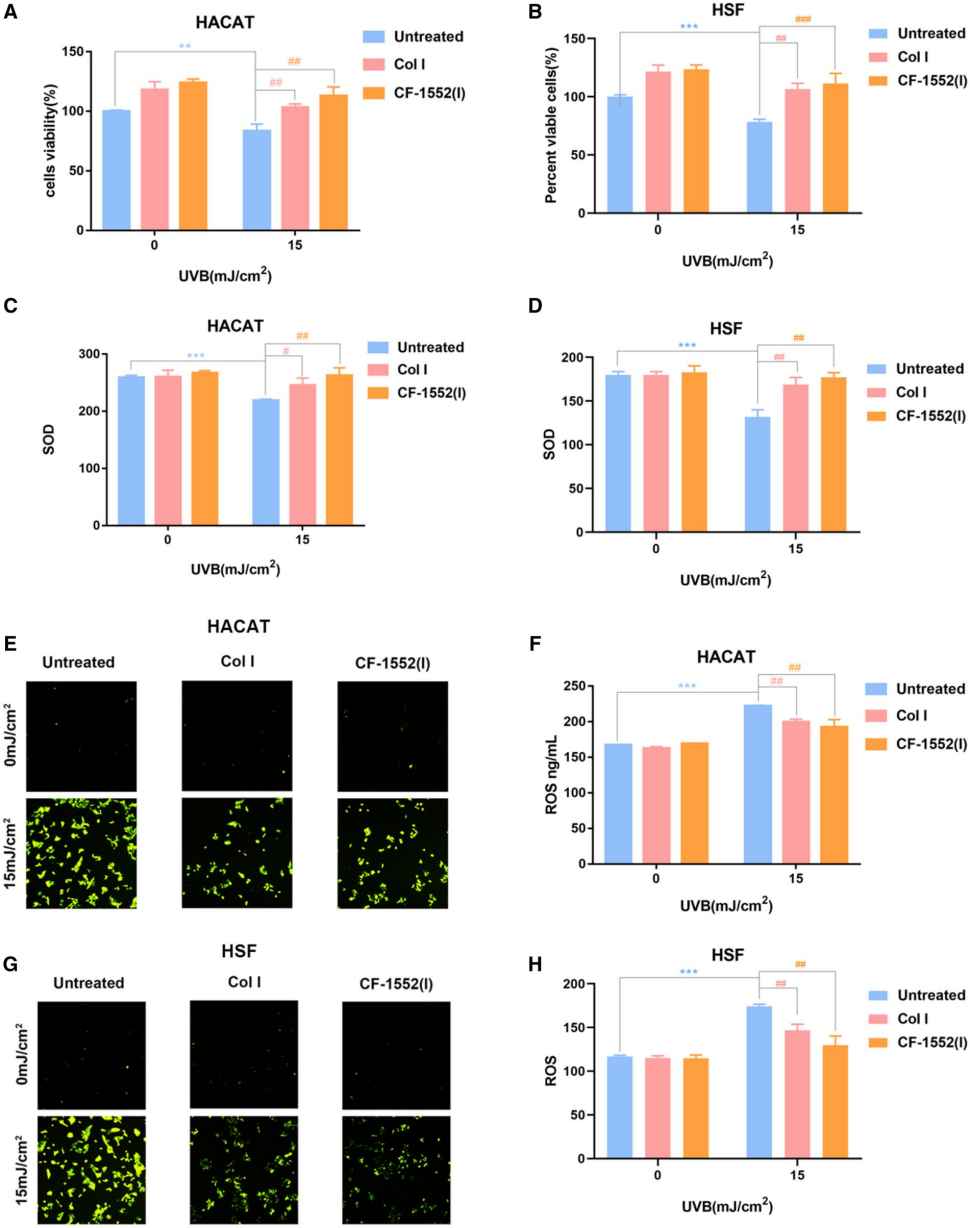
(**A**) Detection of changes in viability of cells (HACAT) treated with different collagen after irradiation (UVB = 15 mJ/cm^2^) compared to cells not irradiated (UVB = 0 mJ/cm^2^) (compared to the normal group: **P <* *0.01, compared to the control group: ##*P *<* *0.01, *n* = 3); (**B**) detection of changes in viability of cells (HSF) treated with different collagen after irradiation (UVB = 15 mJ/cm^2^) compared to cells not irradiated (UVB = 0 mJ/cm^2^) (compared to the normal group: **P <* *0.01, compared to the control group: ##*P *<* *0.01, *n* = 3); (**C**) changes in the content of SOD in cells (HACAT) treated with different collagen after irradiation (UVB = 15mJ/cm^2^) were detected by the kit (compared to the normal group: ***P <* *0.001, compared to the control group: #*P *<* *0.05, ##*P *<* *0.01, *n* = 3); (**D**) changes in the content of SOD in cells (HSF) treated with different collagen after irradiation (UVB = 15 mJ/cm^2^) were detected by the kit (compared to the normal group: ***P <* *0.001, compared to the control group: #*P *<* *0.05, ##*P *<* *0.01, *n* = 3); (**E**) ROS fluorescent probe (DCFH) labeled cells (HACAT) after irradiation (UVB = 15 mJ/cm^2^) to detect intracellular reactive oxygen species damage. The fluorescence indicates the intracellular ROS content; (**F**) intracellular (HACAT) ROS levels after collagen treatment were measured with the ROS kit (compared to the normal group: ***P <* *0.001, compared to the control group: ##*P *<* *0.01, *n* = 3); (**G**) ROS fluorescent probe (DCFH) labeled cells (HSF) after irradiation (UVB = 15 mJ/cm^2^) to detect intracellular reactive oxygen species damage. The fluorescence indicates the intracellular ROS content and (**H**) intracellular (HSF) ROS levels after collagen treatment were measured with the ROS kit (compared to the normal group: ***P <* *0.001, compared to the control group: ##*P *<* *0.01, *n* = 3).

We investigated the impact of collagen on the cytotoxicity of UVB irradiation by assessing cell viability [47, 48]. As depicted in Figure 3A, the viability of UVB-irradiated HACAT cells (15 mJ/cm^2^) dropped to 83.39 ± 5.91%, a significant decrease compared to non-irradiated cells (0 mJ/cm^2^) (**P < 0.01). However, after treatment with Col I, the cell viability increased to 103.21 ± 2.98%, and treatment with CF-1552(I) raised it to 113.07 ± 7.40%, showing a significant improvement compared to the untreated group (##*P* < 0.01). Similarly, Figure 3B illustrates that UVB-irradiated HSF cells (15 mJ/cm^2^) had a viability of 78.41 ± 2.31%, significantly lower than that of non-irradiated cells (**P < 0.01). After Col I treatment, viability increased to 106.74 ± 4.86%, and CF-1552(I) treatment led to 111.36 ± 8.75%, both showing significant enhancements over the untreated cells (##*P* < 0.01). At the cellular level, collagen was found to improve the viability of both HACAT and HSF cells post-UVB irradiation, with no significant differences between the Col I and CF-1552(I) treatments. Collagen exhibited a proliferative effect on both cell types, accelerating cell renewal and enhancing overall viability despite UV-induced damage. Thus, both Col I and CF-1552(I) effectively restored cell viability after UVB irradiation.

#### Effect of Col I and CF-1552(I) on the amount of SOD in HACAT and HSF cells after UVB irradiation

As shown in Figure 3C, UVB irradiation (15 mJ/cm^2^) significantly decreased the intracellular SOD content in HACAT cells compared to non-irradiated cells (0 mJ/cm^2^) (***P < 0.001). When these UVB-irradiated HACAT cells were cultured with a medium containing Col I and CF-1552(I), the intracellular SOD content increased significantly. Notably, cells cultured with CF-1552(I) showed a more pronounced increase in SOD content compared to those cultured with Col I (#*P* < 0.05, ##*P* < 0.01). Similarly, Figure 3D illustrates that UVB irradiation (15 mJ/cm^2^) reduced the intracellular SOD content in HSF cells compared to the non-irradiated controls (0 mJ/cm^2^) (***P < 0.001). UVB-irradiated HSF cells cultured with Col I and CF-1552(I) also exhibited a significant increase in SOD content. Again, cells treated with CF-1552(I) demonstrated a more significant rise in SOD content than those treated with Col I (##*P* < 0.01). Overall, both Col I and CF-1552(I) were effective in increasing SOD expression, with no significant difference between the two treatments in elevating SOD levels in the cells.

#### Effect of Col I and CF-1552(I) on the amount of intracellular ROS in HACAT and HSF cells after UVB irradiation

Figure 3E and F demonstrate that HACAT cells exposed to UVB irradiation (15 mJ/cm^2^) exhibit a significant increase in intracellular ROS levels compared to non-irradiated HACAT cells (0 mJ/cm^2^) (***P < 0.001). When these UVB-irradiated cells were cultured in media containing Col I or CF-1552(I), there was a marked reduction in ROS levels compared to the untreated group (##*P* < 0.01). This suggests that both Col I and CF-1552(I) have a cellular-level reparative effect on photo-aging. Comparison between the Col I and CF-1552(I) treatments indicated no significant difference in their efficacy in reducing ROS levels, implying that both treatments are equally effective in mitigating UVB-induced cellular damage.

Similarly, Figure 3G and H shows that HSF cells subjected to UVB (15 mJ/cm^2^) also demonstrated a significant increase in intracellular ROS compared to those without UVB exposure (0 mJ/cm^2^) (***P < 0.001). Culturing UVB-irradiated HSF cells in media with Col I or CF-1552(I) resulted in a significant reduction in ROS levels compared to the untreated group (##*P* < 0.01), reinforcing the reparative effect of Col I and CF-1552(I) on photo-aging at the cellular level. No significant difference was observed between the two treatments in their impact on ROS reduction, further indicating their equivalent effectiveness in repairing UVB-induced ROS damage.

UVB radiation leads to the excessive production of superoxide anions in both HACAT and HSF cells, disrupting the cellular oxidative balance. The cell’s endogenous antioxidant defenses are insufficient to counteract this ROS overproduction, necessitating an increase in intracellular antioxidant enzyme expression. The SOD assay results show that both Col I and CF-1552(I) enhance intracellular SOD expression and reduce ROS levels. Consequently, Col I and CF-1552(I) are effective in increasing antioxidant enzyme levels and protecting HACAT and HSF cells from oxidative stress induced by UVB radiation. Moreover, there is no significant difference between the two treatments regarding their effectiveness in reducing ROS content.

### Skin wound repair: mouse dorsal skin defect wound model

To assess the effectiveness of Col I and CF-1552(I) in wound repair, we conducted experiments on common wounds on the backs of mice. This included analyzing the wound area, collagen deposition, and gene expression during the healing process to evaluate how collagen influences skin wound repair [49, 50].

#### Wound-healing effect of the Col I and CF-1552(I)

**Figure 4.**
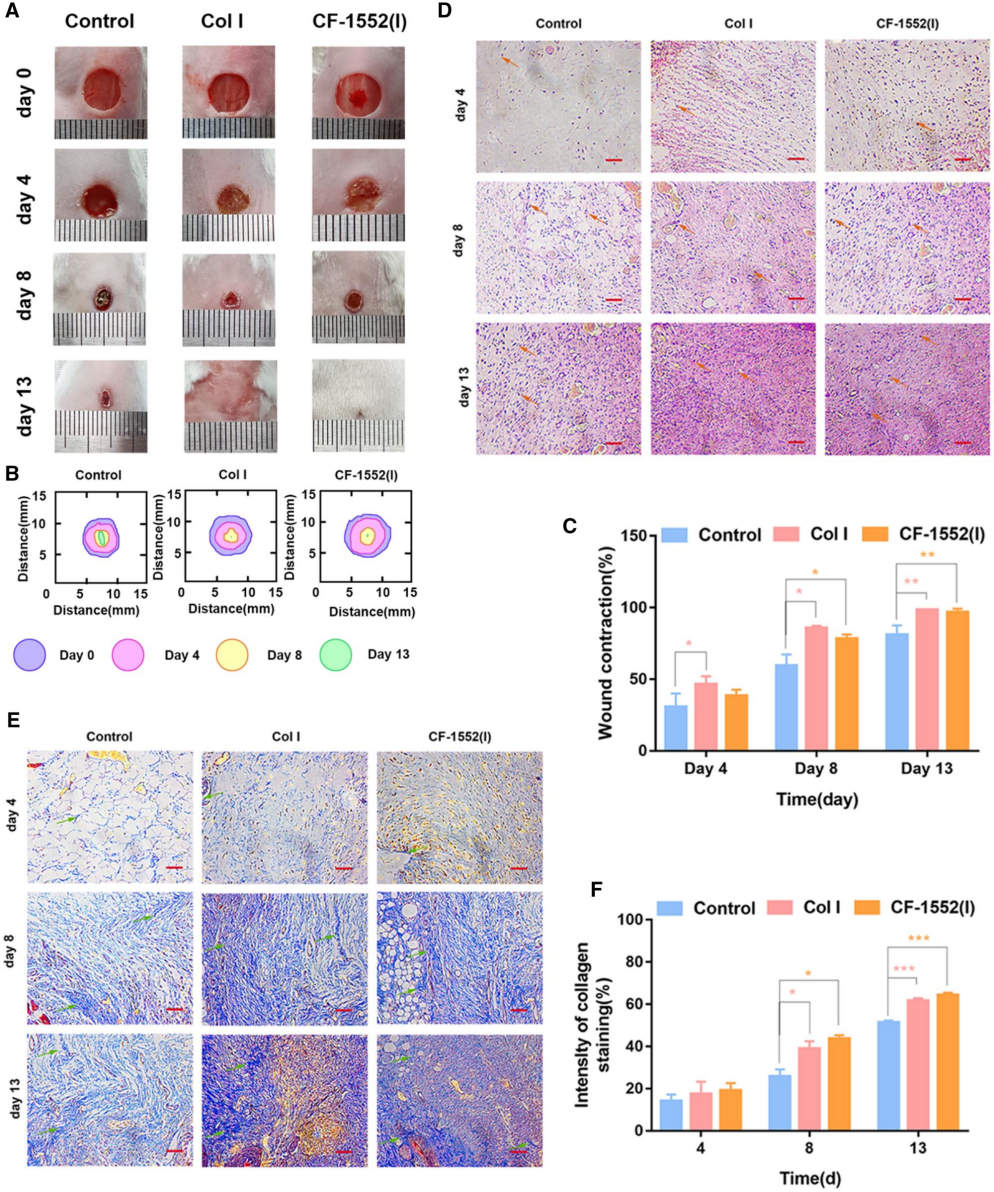
(**A**) Photographs of wounds at days 0, 4, 8 and 13; (**B**) traces of wound-bed closure on days 0, 4, 8 and 13 for each group; (**C**) wound contraction for each group (*P <* *0.05, **P <* *0.01, *n* = 3); (**D**) H&E staining of wounds after 4, 8 and 13 days (blood vessels: arrow indication, scale bar: 100 μm); (**E**) Masson’s trichrome staining of a wound site after 4, 8 and 13 days (collagen fibers: arrow indication, scale bar: 100 μm) and (**F**) analysis of collagen staining intensity (*P <* *0.05, ***P <* *0.001, *n* = 3).

Skin injuries on the backs of mice were used to model ordinary wounds [51], as depicted in Figure 4A and B, with the results of several parallel experiments analyzed in Figure 4C. On day 4, the Col I group exhibited better wound contractility compared to the control group (*P < 0.05). By day 8, the Col I group’s advantage over the control group was more pronounced, and the CF-1552(I) group also demonstrated significant wound contractility. By day 13, wounds in the control group had not fully healed, whereas those in the Col I and CF-1552(I) groups were completely closed (**P < 0.01). This indicates that both Col I and CF-1552(I) were effective in skin wound repair. Notably, the Col I group showed earlier wound repair compared to CF-1552(I), likely because the gene fragment in Col I that promotes early wound repair was absent in CF-1552(I). However, both groups achieved complete wound healing simultaneously, suggesting that the genes in Col I promoting early repair do not influence the final repair outcome.

#### Histological evaluation of wound repair

Collagen deposition at the wound site is a key indicator of wound healing [52]. To assess this, Masson staining was utilized on mouse skin samples collected on days 4, 8 and 13 to visualize new collagen formation. As illustrated in Figure 4E and F, the results revealed significantly higher levels of *de novo* collagen deposition in the Col I and CF-1552(I) groups compared to the control group on both day 8 and day 13. There was no significant difference between the Col I and CF-1552(I) groups (*P < 0.05, ***P < 0.001), suggesting that both treatments were equally effective in promoting collagen deposition and accelerating wound healing.

Granulation tissue abundance is a key indicator of wound healing, and H&E staining was used to assess its presence in the skin of mice on days 4, 8, and 13. As shown in Figure 4D, both the Col I and CF-1552(I) groups exhibited fewer inflammatory cells by day 8 and greater angiogenesis by day 13 compared to the control group. The effects of Col I and CF-1552(I) on reducing inflammation and promoting angiogenesis were similar. Collagen, known to enhance VEGF gene expression [53], influences various wound-healing processes such as angiogenesis, re-epithelialization, and collagen synthesis [54]. Consequently, both Col I and CF-1552(I) effectively promoted angiogenesis and collagen deposition, with no significant difference between the two in terms of their efficacy.

#### α-SMA, Col I and Col III expression in wound regeneration with various treatment

**Figure 5.**
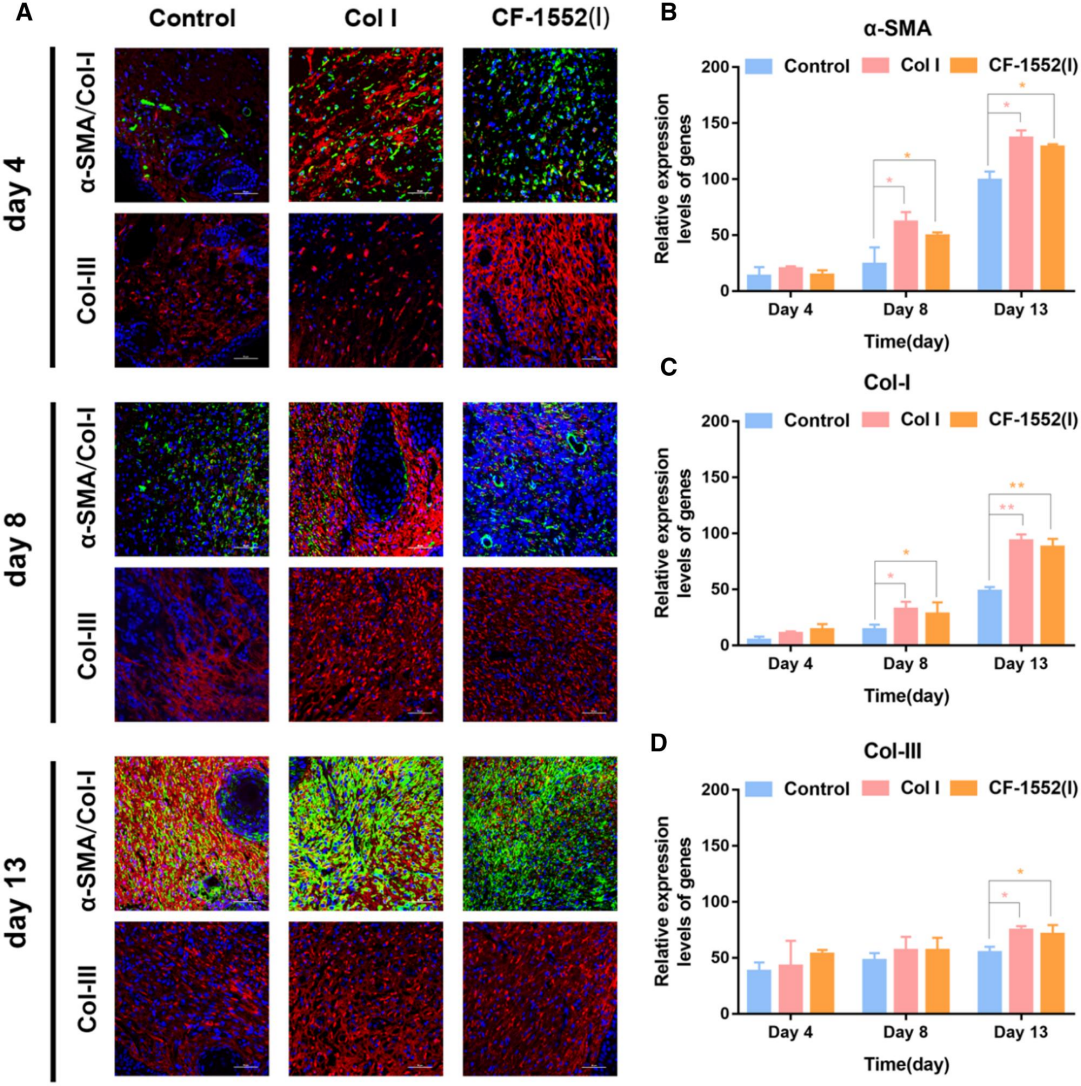
(**A**) Images of immunofluorescence labeling of skin wound tissues with α-SMA, Col I and Col III (scale bar: 100 μm); (**B**) α-SMA quantitative analysis of immunofluorescence images (*n* = 3); (**C**) Col I quantitative analysis of immunofluorescence images (*P <* *0.05, *n* = 3) and (**D**) Col III quantitative analysis of immunofluorescence images (*P <* *0.05, **P <* *0.01, *n* = 3).

To further investigate the biological mechanism of collagen in skin wound healing, we quantitatively analyzed the expression of α-SMA, Col I, and Col III [55, 56]. As illustrated in Figure 5A and B, both the Col I and CF-1552(I) groups significantly increased α-SMA protein expression compared to the control group on days 8 and 13 (*P < 0.05). Similarly, Figure 5A and C shows that both Col I and CF-1552(I) groups notably enhanced Col I protein expression on these days (*P < 0.05, **P < 0.01). Furthermore, Figure 5A and D indicates that on day 13, Col I and CF-1552(I) groups significantly raised Col III protein levels compared to the control group (*P < 0.05). Both Col I and CF-1552(I) groups had comparable effects in promoting α-SMA, Col I, and Col III protein expressions, which suggests that they enhance wound healing by upregulating the TGF-β1 gene and increasing these proteins. Thus, these findings demonstrate that Col I and CF-1552(I) significantly accelerate skin wound repair, with no substantial difference observed between the two treatments in their effects on α-SMA, Col I, and Col III protein expression during the healing process.

### Skin photo-aging repair: UVB irradiation photo-aging model of mouse dorsal skin

In order to further investigate the repairing efficacy of Col I and CF-1552(I) on UVB irradiation-induced skin aging, we conducted a collagen-repairing experiment on photo-aged skin on the back of mice, as well as the determination of related factors to verify the repairing effect of collagen on UVB-induced skin aging.

#### Non-invasive method for evaluating changes in skin condition

**Figure 6.**
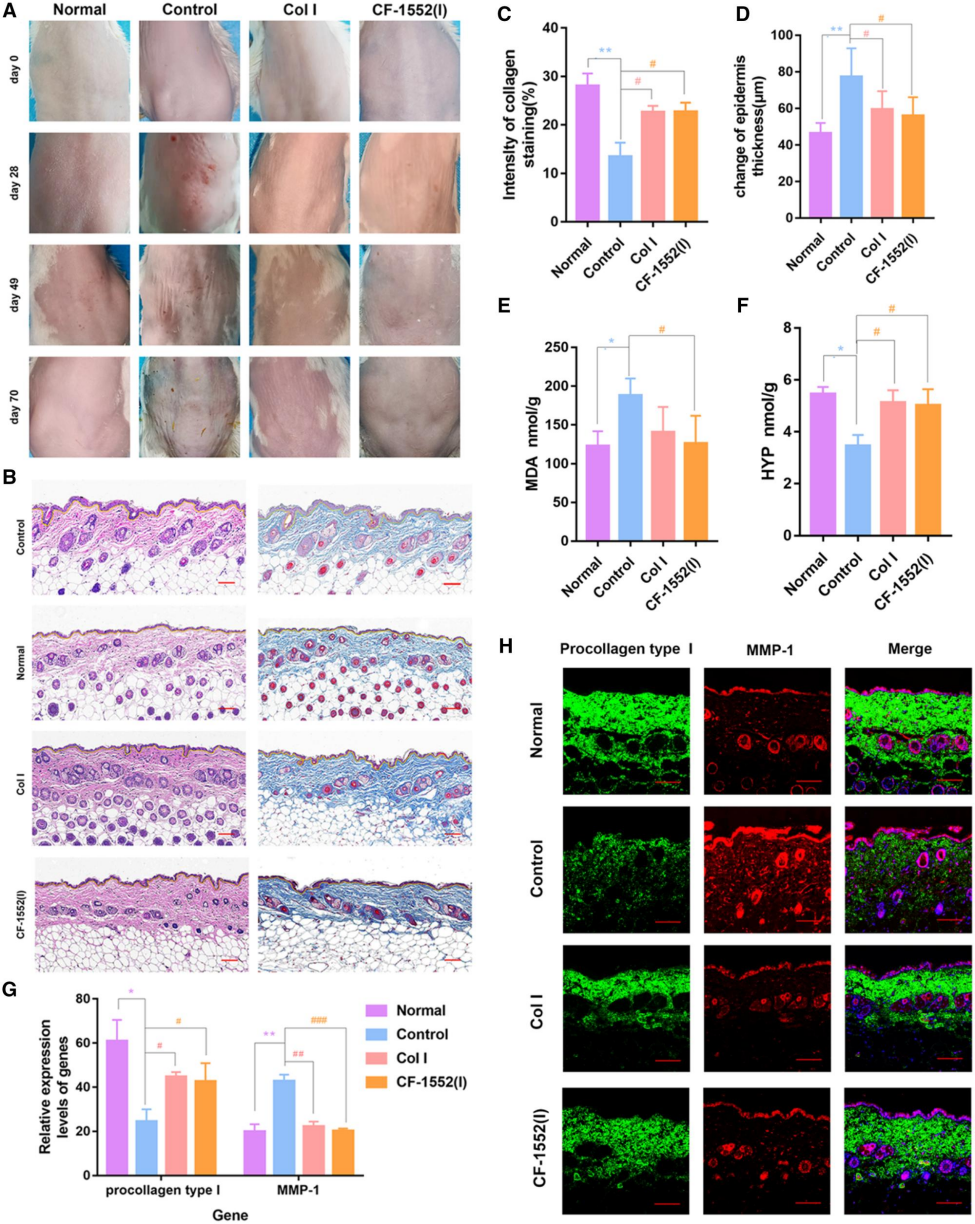
(**A**) The appearance of folds in the dorsal skin of UVB-irradiated mice at days 0, 28, 49 and 70 (UVB = 0.39 J/cm^2^); (**B**) H&E and Masson’s tricolor staining of the back skin after 70 days (dermis: yellow lines, scale bar: 100 μm); (**C**) analysis of collagen staining intensity (compared to the normal group: **P <* *0.01, compared to the control group: #*P *<* *0.05, *n* = 3); (**D**) statistical chart of epidermal thickness (compared to the normal group: **P <* *0.01, compared to the control group: #*P *<* *0.05, *n* = 3); (**E**) detection of MDA in skin tissue with the kit (compared to the normal group: *P <* *0.05, compared to the control group: #*P* < 0.05, *n* = 3); (**F**) detection of HYP in skin tissue with the kit (compared to the normal group: **P <* *0.01, compared to the control group: #*P *<* *0.05, *n* = 3); (**G**) day 70 quantitative analysis of immunofluorescence images (compared to the normal group: *P <* *0.05, **P <* *0.01, compared to the control group: #*P *<* *0.05, ##*P *<* *0.01, ###*P *<* *0.001, *n* = 3) and (**H**) images of immunofluorescence labeling of skin wound tissues with procollagen type I and MMP-1 on day 70 (scale bar: 100 μm).

Non-invasive assessment methods, such as observing skin fold production, minimize damage to the animals’ skin [57, 58]. As shown in Figure 6A, irregular wrinkles on the back skin of mice were clearly evident in the control group compared to the normal group at week 10. This wrinkle formation, characterized by a loss of smoothness, aligns with the description of photo-aging in the literature, confirming the successful establishment of an animal model for skin damage. In contrast, the group treated with Col I and CF-1552(I) showed a reduction in the number of wrinkles on the dorsal skin compared to the control group, indicating that both Col I and CF-1552(I) are effective in mitigating wrinkles caused by UVB irradiation-induced skin aging.

**Figure 7.**
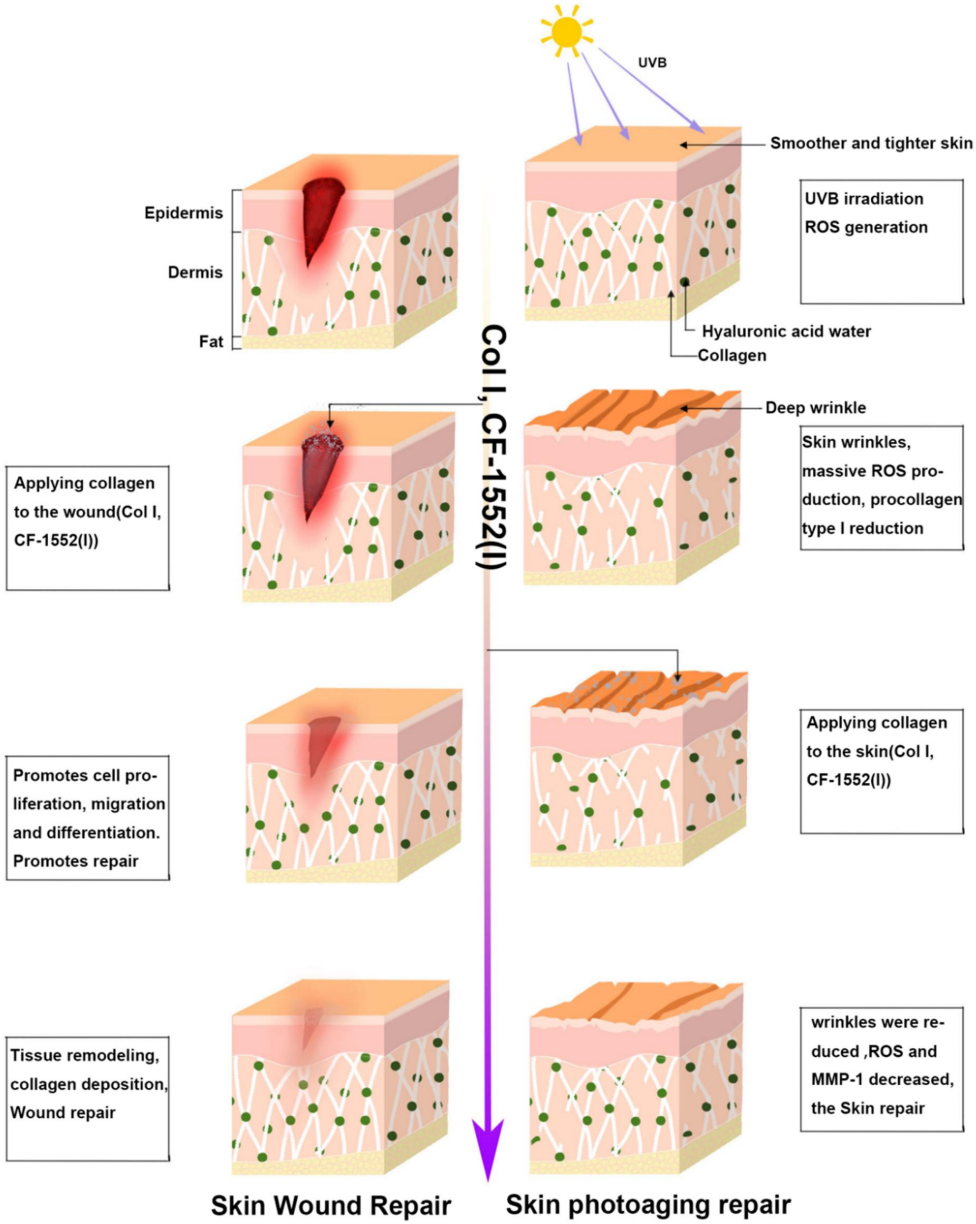
Skin wound repair and skin photo-aging repair mechanisms.

#### Effect of Col I and CF-1552(I) on MDA and HYP levels

Exposure of skin to UVB radiation increases ROS levels, which leads to reduced SOD activity, disruption of physiological homeostasis and elevated MDA levels due to accelerated lipid peroxidation [59–61]. To assess the degree of photo-aging and the efficacy of Col I and CF-1552(I) in skin repair, MDA content in mouse skin tissues was measured. As shown in Figure 6E, MDA levels in UVB-irradiated skin tissues of the control group (0.39 J/cm^2^) were significantly higher (*P < 0.05) compared to the normal group (0 J/cm^2^) which was not irradiated. Following collagen coating with either Col I or CF-1552(I) in some control group mice, MDA levels in the CF-1552(I) group were significantly reduced, almost returning to baseline levels compared to the control group (#*P* < 0.05). Although MDA levels also decreased in the Col I group compared to the control group, the difference was not significant. This suggests that both Col I and CF-1552(I) help mitigate UV-induced ROS increase by enhancing SOD activity, thereby reducing lipid peroxidation and MDA levels. However, CF-1552(I) proved to be more effective than Col I in reducing MDA content, likely due to its rapid degradation into peptides and the increased permeability of active ingredients through the skin. To study the biological activity of CF-1552(I) degradation products, we hydrolyzed CF-1552(I) with pepsin and trypsin, following established research methods [62]. The enzymes were inactivated by heating the mixture in a boiling water bath for 10 min, then centrifuging at 25°C and 10 000g for 10 min. The supernatant was collected, and the degradation products were isolated using a dialysis bag with a molecular weight cutoff of 100–500 Da (Solarbio, YA1069). SDS-PAGE analysis showed that the degraded CF-1552(I) had a molecular weight of about 10 kDa (Supplementary Figure S1a). We tested the effects of 1 mg/ml degradation products on the proliferation of HACAT and HSF cells and found that they significantly enhanced cell proliferation compared to CF-1552(I) (*P* < 0.01) (Supplementary Figure S1b).

The antioxidant properties of collagen may stem from its high content of glycine (G), proline (P) and charged amino acid residues, such as arginine (R) [63, 64]. In our study, CF-1552(I), which is rich in G, P and R, effectively captures and eliminates ROS. Therefore, it can be inferred that CF-1552(I) and its degradation products have strong antioxidant properties in comparison to CF-1552(I).

Given CF-1552(I)’s superior MDA reduction effect over Col I, we hypothesized this might be due to its degradation products. Thus, we focused on their antioxidant properties. At 1 mg/ml, the degradation products exhibited higher antioxidant activity than CF-1552(I), as evidenced by greater DPPH scavenging efficiency (*P* < 0.05) (Supplementary Figure S1c). Additionally, the degradation products improved UVB-induced oxidative stress in HACAT and HSF cells, restoring SOD levels more effectively than CF-1552(I) at the same concentration and significantly reducing MDA content (Supplementary Figure S1d and e). These findings indicate that the antioxidant effect of CF-1552(I) degradation products is comparable to CF-1552(I) itself, aligning with numerous studies reporting enhanced antioxidant and biological activity of collagen peptides post-degradation [65–67]. A more comprehensive study on the degradation products of CF1552 will be conducted in our future research.

Hydroxyproline (HYP), an important indicator of skin aging, is a key amino acid in collagen and serves as a measure of collagen content in the dermis [68]. To assess the extent of photo-aging and the effectiveness of Col I and CF-1552(I) in skin repair, we measured HYP levels in mouse skin tissues. As depicted in Figure 6F, UVB irradiation (0.39 J/cm^2^) led to a significant reduction in HYP content compared to non-irradiated controls (0 J/cm^2^) (**P < 0.01). However, in UVB-irradiated mice, treatment with Col I and CF-1552(I) resulted in increased HYP levels compared to the control group, with both treatments showing positive effects (#*P* < 0.05). Under normal conditions (UVB = 0 J/cm^2^), collagen does not affect HYP levels. Overexpression of elastase disrupts collagen and elastin in the ECM, but Col I and CF-1552(I) enhance HYP expression by downregulating elastase. Our results indicate that both Col I and CF-1552(I) effectively boost HYP content in photo-aged skin, with no significant difference in their enhancement capabilities.

#### Histological evaluation of the skin

Epidermal thickening, a hallmark of photo-aging, was assessed using H&E staining across various groups of representative skin tissues to evaluate epidermal and dermal thicknesses [69]. As depicted in Figure 6B and D, the control group exhibited a notable increase in epidermal thickness following UVB (0.39 J/cm^2^) irradiation compared to the normal group (*P < 0.05, **P < 0.01). Conversely, treatment with Col I and CF-1552(I) resulted in a thinner epidermal layer relative to the control group. Prolonged UVB exposure also led to a reduction in collagen content within the skin, which was measured using Masson staining. As shown in Figure 6B and C, collagen content in the UVB-irradiated control group significantly decreased compared to the normal group without UVB exposure (0 J/cm^2^). However, treatment with Col I and CF-1552(I) restored collagen levels to near-normal conditions. The effectiveness of these treatments was consistent across both Col I and CF-1552(I) groups. Increased ROS due to UV irradiation alters dermal connective tissue proteins, contributing to thicker skin and reduced collagen deposition [70]. Col I and CF-1552(I) effectively lower ROS levels, thereby improving skin thickness and collagen content. Additionally, there were no significant differences between Col I and CF-1552(I) regarding their effects on skin thickness recovery and collagen deposition post-UV irradiation.

#### Expression of procollagen type I and MMP-1 in skin repair under different treatment conditions

Matrix metalloproteinases (MMPs), including MMP-1, are crucial in destructive processes such as inflammation, tumor invasion, and skin aging. Specifically, MMP-1, or mesenchymal collagenase, begins the breakdown of type I, II, and III collagen in the skin [71, 72]. UVB irradiation increases MMP-1 expression in normal human skin while decreasing procollagen type I levels. Our study measured the expression of MMP-1 and procollagen type I under UVB exposure (0.39 J/cm^2^) [73]. As depicted in Figure 6G and H, UVB exposure significantly elevated MMP-1 expression and reduced procollagen type I in the control group compared to the normal group (**P < 0.01 for MMP-1 and *P < 0.05 for procollagen type I). Treatment with Col I and CF-1552(I) led to a decrease in MMP-1 levels and a restoration of procollagen type I expression compared to the control group, with CF-1552(I) showing a more pronounced effect on MMP-1 inhibition (#*P* < 0.05, ##*P* < 0.01). Both Col I and CF-1552(I) effectively repaired UV-induced skin damage by inhibiting MMP-1 and restoring procollagen type I. There was no significant difference in the efficacy of Col I and CF-1552(I) in restoring procollagen type I levels after UV irradiation.

In conclusion, a study of recombinant type I collagen with CF1552 to mitigate UV ageing and promote skin repair was carried out. After UVB irradiation, the skin skin is damaged and generates oxidative stress that is difficult to repair on its own, and CF1552 with COlI promotes skin regeneration by facilitating collagen deposition, scavenging excessive ROS, and decreasing the expression of MMP1 (Figure 7).

## Conclusion

Recombinant collagen, known for its biocompatibility, stability, controlled quality, and low viral risk, was investigated for its effectiveness in skin repair. In the field of skin tissue engineering, a lot of research has been carried out on collagen materials for skin repair and their potential applications. Supplementary Table S1 describes the studies carried out by various researchers using different sources of collagen as a key ingredient in different formulations (e.g. fish skin collagen, collagen cream, collagen peptide and collagen hydrogel). Through their study, the authors observed the effective wound-healing activity of fish skin collagen from Nile tilapia skin. The results showed that the wound healing rate was 38.8% ± 22.8% in the fish skin collagen-treated group and 8.7% ± 17.2% in the fish skin collagen-treated group, whereas the *in vitro* scratch healing rate of fish skin collagen was significantly higher than that of the model control group at a concentration of 50.0 μg/ml. The PSD18 wound-healing rate in the control group was 72.1% ± 13.9%, and the best wound-healing was achieved with fish skin collagen. In addition, histological assessment showed that fish skin collagen promoted new epidermal layer formation, proliferation, fibroblast formation and new capillarization compared to the model control group [74]. Wounds severely affect the external skin barrier. Further research describes a new approach using bovine collagen cream to promote wound healing. In this study, researchers used the NIH 3T3 fibroblast cell line showing increased cell viability without toxicity [75]. Another study reported the role of collagen peptides isolated from jellyfish in wound healing. The results showed that collagen peptide-treated cells exhibited significant scratch closure at 18, 36 and 48 h at a concentration of 6.25 μg/ml compared to the control group. In *in vivo* studies, rapid contraction of whole-layer wounds, followed by an increase in collagen deposition and fibroblast growth factor, has been fruitful [76]. In order to accelerate the wound-healing process, the authors reported the combination of guanosine tetrahedral hydrogel with recombinant human-derived collagen (G4-RHC). The obtained hydrogel had dermatogenic wound-healing properties. *In vitro* wound-healing experiments with L929 cells treated with G4-RHC hydrogel showed that there was rapid migration of L929 cells after G4-RHC hydrogel treatment as compared to the control group. The RHC caused fibroblasts and macrophages to turn over toward the wound, which initiated proliferation and migration [77]. Thickening of skin damage and even dermatitis caused by long-term exposure to UV rays is also an urgent problem in the field of skin tissue engineering. Through their research, the authors designed recombinant type III collagen rhCol III with significant antioxidant capacity by synthetic biology technology, and the results of the eighth week of experiments showed that it could reduce skin photo-aging caused by UV radiation, including reducing the thickening of the epidermis and dermis. The results show that rhCol III can reduce UV-induced photo-damage, including reduction of epidermal and dermal thickening, increase of Col I and Col III secretion, and extracellular mesenchymal remodeling (ECM) [69]. High-purity collagen was extracted from porcine skin, the proliferation rate of HACAT and HSF cells treated with porcine skin collagen was not significantly different from that of control cells, and the number of migrating cells was increased by about 40% at a collagen concentration of 0.6 mg/ml compared with the control group, and the proportion of Col I and Col III fibers was significantly increased after 1 month of injection of porcine skin collagen to delay the aging of the skin [78]. In one study, non-denatured type I collagen (YCI) was extracted from yak skin, the relative proliferation rate of HSFs (HFF-1) in 0.1-mg/ml YCI solution gradually increased to 120% on day 3 and 150% on day 5, which indicated that YCI significantly promoted the proliferation of HFF-1, and the content of HYP increased to 106.13% after the treatment of YCI, which indicated that the dermal collagen content increased, and the proportion of migrating cells increased significantly after injection of pig skin collagen for 1 month to delay skin aging [6]. The increase in dermal collagen content and the significant recovery of collagen content to normal levels within 4 days indicated that YCI has the ability to promote dermal collagen regeneration and accelerate the healing of severe UV-mediated skin damage [79]. Huang et al. obtained collagen polypeptide (SCSCP) with collagenase inhibitory activity from silver carp (hypophthalmichthys molitrix) skin by optimizing the enzymatic process, and SCSCP at a concentration of 10 mg/ml possessed high DPPH and hydroxyl radical scavenging activities, whereas 0.1 mg/ml SCSCP significantly restored UVB-induced viability of L929 cells and inhibited UVB irradiation after L929 cell MMP-1 secretion after UVB irradiation to achieve anti-photo-aging effects [80].

Although collagen and its derivatives from a variety of different sources have been used in the field of skin damage repair. However, almost all collagen used for biomedical and pharmaceutical applications and tissue-engineered medical products is primarily of animal origin [81]. However, safety factors should be considered when using animal-derived collagen, including the risk of transmitting viruses and diseases, pathogenicity, and allergic reactions [82].

We preferred to produce the full-length or fragment of the functional amino acid sequence encoded by the human type I collagen gene by synthetic biology techniques, so that the amino acid sequences of Col I and CF-1552 are identical to those of natural collagen, and this purely natural amino acid sequence does not include any recognition site for the discoidal structural domain receptor, and does not affect MMPs (including membrane type 1 (MT1-MMP), MMP1 and MMP2) expression [83, 84] and is a promising starting material for tissue-engineered medical product matrixes.

In this study, both Col I and CF-1552(I) collagen materials were simultaneously evaluated for their efficacy in wound repair and photo-aging skin repair. The results demonstrated that both types of collagen effectively promote skin wound healing and mitigate photo-aging. Compared with Col I, CF-1552(I), as a functionally enhanced small molecule collagen, has shown comparable skin repair performance in some experiments, indicating that CF-1552(I) has a more promising future in skin repair.

## Supplementary Material

rbae108_Supplementary_Data
