# Correction to: Recombinant collagen for the repair of skin wounds and photo-aging damage

**DOI:** 10.1093/rb/rbaf064

**Published:** 2025-06-29

This is a correction to: Taishan Liu, Jiayun Hao, Huan Lei, Yanru Chen, Lin Liu, Liping Jia, Juan Gu, Huaping Kang, Jingjing Shi, Jing He, Yangbin Song, Yuqi Tang, Daidi Fan, Recombinant collagen for the repair of skin wounds and photo-aging damage, *Regenerative Biomaterials*, Volume 11, 2024, rbae108, https://doi.org/10.1093/rb/rbae108

The authors regretfully discovered that there are errors in Figure 4. The HE images of the Control and CF1552 (I) groups on day 8 in Figure 4D were used incorrectly, and the Masson images of the COLI group on day 8 and the Control group on day 13 in Fig.4E were used incorrectly. This error was caused by confusion in the folder naming during the image classification process.

The Masson quantification in Figure 4F was intended to semi-quantitatively highlight the differences in collagen deposition between the different groups, and the positive rates of each group were originally calculated based on the original images, and the results remain unchanged. This correction does not affect the conclusions of this article.

The corrected version of the Figure is shown below:

**Figure 4.**
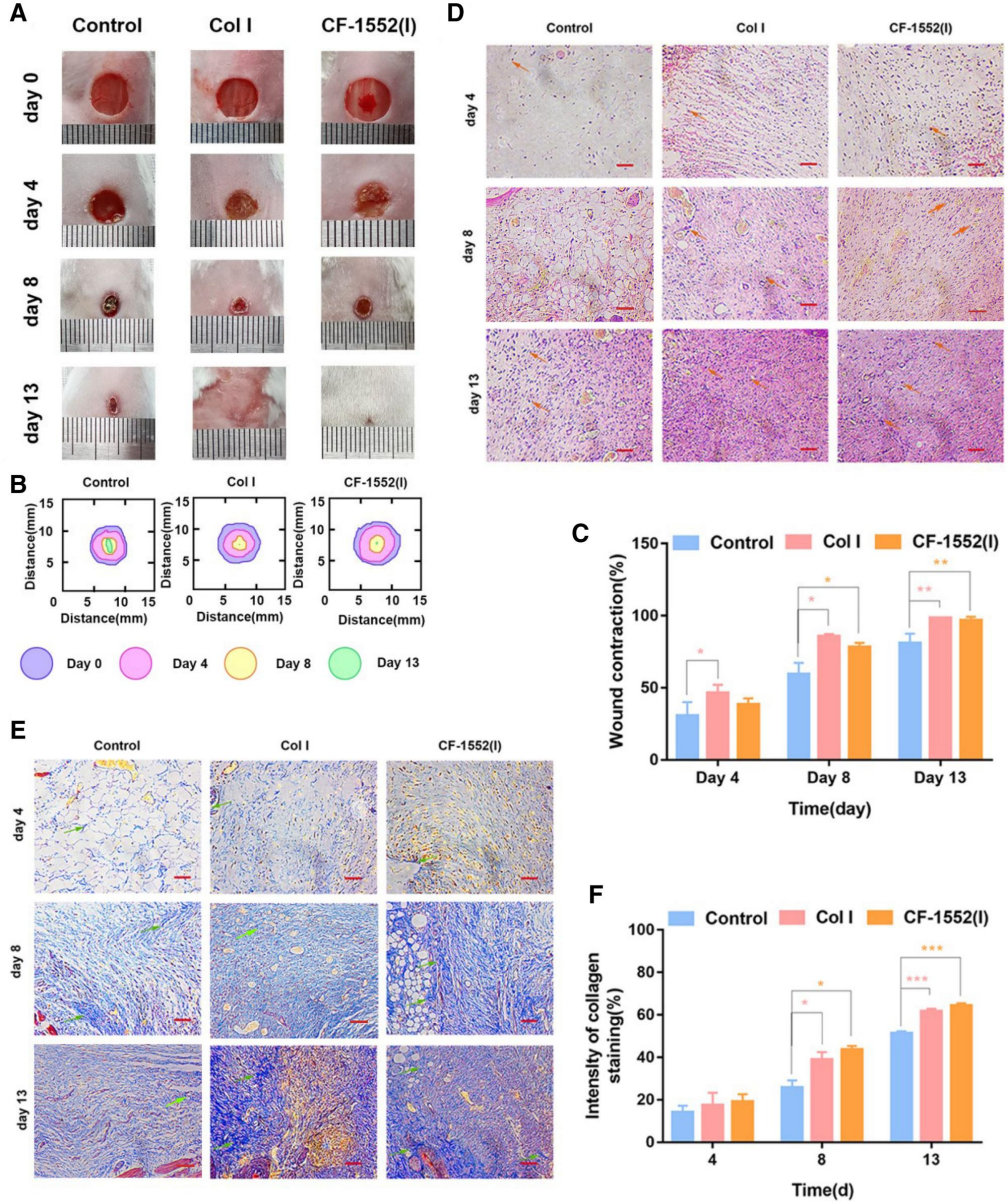
A) Photographs of wounds at days 0, 4, 8, and 13; B) Traces of wound-bed closure on days 0, 4, 8 and 13 for each group; C) Wound contraction for each group. (*p < 0.05, **p < 0.01, n = 3); D) H&E staining of wounds after 4, 8 and 13 days. (blood vessels: orange arrows, scale bar: 100 μm); E) Masson’s trichrome staining of a wound site after 4, 8 and 13 days (Collagen fibers: green arrows, scale bar: 100 μm); F) Analysis of collagen staining intensity. (*p < 0.05, ***p< 0.001, n = 3)

