# Supplementary material for: Recombinant collagen for the repair of skin wounds and photo-aging damage
Source: Regen Biomater. 2024 Sep 2;11:rbae108. doi: 10.1093/rb/rbae108 (PMC11422187; doi:10.1093/rb/rbae108)
Supplement: rbae108_Supplementary_Data [file rbae108_supplementary_data.zip › manuscript to Reviewer.docx]

**Recombinant collagen for the repair of skin wounds and photo-aging damage**

Taishan Liu^#,1,2,3^，Jiayun Hao ^#,1,2,3^，Huan Lei^#,1,2,3^，Yanru Chen^1,2,3^，Lin Liu^4^，Liping Jia^4^，Juan Gu^5^，Huaping Kang^5^，Jingjing Shi^5^，Jing He^4^，Yangbin Song^5^，Yuqi Tang^5^，Daidi Fan^*,1,2,3^

^1^Shaanxi Key Laboratory of Degradable Biomedical Materials, School of Chemical Engineering, Northwest University, Taibai North Road 229, Xi’an 710069, China.

^2^Shaanxi R&D Center of Biomaterials and Fermentation Engineering, School of Chemical Engineering, Northwest University, Xi’an 710069, China.

^3^Biotech & Biomed Research Institute, Northwest University, Xi’an 710069, China.

^4^Xi’an Giant Biotechnology Co.LTD, Shanglinyuan Road, Xi’an 710100, China.

^5^Shaanxi Giant Biotechnology Co.LTD, Jinye Road, Xi'an, 710076, China.

^#^These authors contributed equally to this work

^*^Corresponding Author

Daidi Fan (Corresponding Author, ORCID: 0000-0001-9798-1674)

E-mail: [fandaidi@nwu.edu.cn](mailto:fandaidi@nwu.edu.cn)

Abstract

The skin, being the body's primary defense mechanism, is susceptible to various injuries such as epidermal wounds, natural aging, and ultraviolet-induced damage. As a result, there is growing interest in researching skin repair methods. Traditional animal-derived collagen, widely available on the market, poses risks due to its immunogenicity and potential for viral contamination. In contrast, recombinant collagen sourced from human genes offers a safer alternative. To investigate the potential of human recombinant collagen in skin repair, our research team applied two types, Col I and CF-1552(I), to two different skin injury models: a wound healing model and a photo-aging model. Our findings indicate that both Col I and CF-1552(I) effectively enhance wound healing and repair skin damaged by ultraviolet exposure. Notably, CF-1552(I) showed effects comparable to Col I in promoting cell proliferation in the wound healing model and increasing MDA content in the photo-aging model, suggesting that CF-1552(I) may offer greater potential for skin repair compared to the larger Col I molecule.

Keywords: recombinant collagen, wound repair, skin photo-aging repair, cell proliferation.

1 Introduction

The skin, the largest organ in the human body, functions as the main interface with the external environment. It plays a crucial role in regulating body temperature, sensing pain and stress, defending against pathogens, and protecting against ultraviolet (UV) radiation [1, 2]. As the body's primary defense mechanism, the skin is constantly exposed to daily damage from trauma, bacterial infections, burns, natural aging, and UVB-induced aging [3]. This ongoing and intricate process of skin repair underscores its significant value [4, 5].

Collagen is extensively utilized in skin repair research due to its significant biological properties as an extracellular matrix component in the human body. It plays a crucial role in supporting the normal physiological functions of cells, tissues, and organs, and it positively affects trauma repair, skin regeneration, and photo-aging recovery [6-8]. Currently, animal-derived collagen, mainly bovine collagen, is predominantly used in biomedical, pharmaceutical, and tissue-engineered medical applications. However, this reliance on animal collagen raises several safety concerns, including risks of pathogenicity, virus transmission, diseases, and allergic reactions[9, 10]. Specifically, transmissible spongiform encephalopathy (TSE), such as bovine spongiform encephalopathy (BSE), poses a significant restriction on the use of bovine collagen from infected regions[11]. Additionally, traditional treatments for animal collagen, like heat treatment, often fail to preserve its structural and functional integrity, further complicating its use[12, 13]. Consequently, these issues highlight the limitations of traditional collagen extraction methods, impeding progress in collagen research and development [14-16].

These phenomena highlight that animal-derived collagen still presents biosafety concerns, underscoring the need for a safe and reliable alternative that is both biocompatible and biologically effective[17]. Our previous research led to the development of recombinant type I human collagen (Col I), which addresses biosafety issues by eliminating the risk of animal-borne infectious diseases and demonstrates enhanced bio-effectiveness with excellent biocompatibility.[18-20]. Concurrently, our team employed DNA recombinant technology to create recombinant type I humanized collagen(CF-1552(I)) by expressing the functional amino acid sequence fragment of human collagen. This humanized collagen not only maintains the characteristics of conventional collagen but also boasts improved water solubility, reduced immune rejection, and superior biological compatibility[21, 22]. CF-1552(I) is derived from the human type I collagen gene chain, but differs from Col I, which encompasses the entire gene sequence of the type I collagen α1(I) chain and closely mirrors natural type I collagen. In contrast, CF-1552(I) is based on a 15-amino-acid fragment of the type I collagen gene, which, after codon optimization and splicing recombination, results in a new recombinant humanized collagen sequence.[23, 24]. The smaller molecular weight of CF-1552(I) compared to Col I enhances its water solubility and enables rapid degradation into a single helix structure suitable for injury site applications. Furthermore, CF-1552(I) has optimized the -(GXY) sequence of the α(I) peptide chain and removed amino acid residues like tryptophan that could cause immune reactions, further minimizing allogeneic rejection. The unique repetitive structure of CF-1552(I), due to its amino acid sequence, allows chemically active amino acids to bind precisely to their target sites, significantly enhancing collagen flexibility.

This study explores the efficacy of Col I and CF-1552 in promoting wound healing and repairing UV-induced skin photo-aging. It also examines the mechanisms by which Col I and CF-1552(I)—a repetitive 15-amino-acid sequence in type I collagen—facilitate these processes. The unique repeatability of CF-1552(I) may enhance skin repair by leading to a higher content of effective gene fragments post-hydrolysis. Additionally, CF-1552(I) exhibits excellent water solubility, making it easier to degrade into peptides that penetrate the skin more effectively and act within cells. The study also focuses on how CF-1552(I) may improve the activity of certain intracellular genes.

2 Materials and Methods

2.1 Materials

Recombinant type I human collagen (Col I) (Presented by Shaanxi Giant Biotechnology Co.LTD, Mw: 110 kDa, ).Recombinant type I humanized collagen (CF-1552(I)) (Presented by Shaanxi Giant Biotechnology Co.LTD, [HPLC] ≥ 99%, Mw: 97 kDa,). The amino acid sequence of COLI and CF1552 were shown in Table 1. DMEM was purchased from HyClone (Logan, UT, USA). Methylthiazolyldiphenyl tetrazolium bromide (MTT) was purchased from Beyotime Biotechnology (Shanghai, China).Bovine Collagen (BC) (Beijing, China). Trypsin and penicillin–streptomycin (pen–strep) were purchased from Solarbio Science & Technology Co., Ltd. (Beijing, China). Fetal bovine serum (FBS) was purchased from Biological Industries (Cromwell, CT, USA). Human skin fibroblasts(HSF) and human immortalized keratinocytes (HACAT) were obtained from Fu Heng Biology (Shanghai, China).

Table 1. The amino acid sequence of COLI and CF1552

| Collagen Types | sequence |
| --- | --- |
| Col I | QLSYGYDEKSTGGISVPGPMGPSGPRGLPGPPGAPGPQGFQGPPGEPGEPGASGPMGPRGPPGPPGKNGDDGEAGKPGRPGERGPPGPQGARGLPGTAGLPGMKGHRGFSGLDGAKGDAGPAGPKGEPGSPGENGAPGQMGPRGLPGERGRPGAPGPAGARGNDGATGAAGPPGPTGPAGPPGFPGAVGAKGEAGPQGPRGSEGPQGVRGEPGPPGPAGAAGPAGNPGADGQPGAKGANGAPGIAGAPGFPGARGPSGPQGPGGPPGPKGNSGEPGAPGSKGDTGAKGEPGPVGVQGPPGPAGEEGKRGARGEPGPTGLPGPPGERGGPGSRGFPGADGVAGPKGPAGERGSPGPAGPKGSPGEAGRPGEAGLPGAKGLTGSPGSPGPDGKTGPPGPAGQDGRPGPPGPPGARGQAGVMGFPGPKGAAGEPGKAGERGVPGPPGAVGPAGKDGEAGAQGPPGPAGPAGERGEQGPAGSPGFQGLPGPAGPPGNDGAKGDAGAPGAPGSQPGEAGKPGEQGVPGDLGAPGPSGARGERGFPGERGVQGPPGPAGPRGANGAGAPGLQGMPGERGAAGLPGPKGDRGDAGPKGADGSPGKDGVRGLTGPIGPPGPAGAPGDKGESGPSGPAGPTGARGAPGDRGEPGPPGPAGFAGPPGADGQPGAKGEPGDAGAKGDAGPPGPAGPAGPPGPIGNVGAPGAKGARGSAGPPGATGFPGAAGRVGPPGPSGNAGPPGPPGPAGKEGGKGPRGETGPAGRPGEVGPPGPPGPAGEKGSPGADGPAGAPGTPGPQGIAGQRGVVGLPGQRGERGFPGLPGPSGEPGKQGPSGASGERGPPGPMGPPGLAGPPGESGREGAPGAEGSPGRDGSPGAKGDRGETGPAGPPGAPGAPGAPGPVGPAGKSGDRGETGPAGPAGPVGPVGARGPAGPQGPRGDKGETGEQGDRGIKGHRGFSGLQGPPGPPGSPGEQGPSGASGPAGPRGPPGSAGAPGKDGLNGLPGPIGPPGPRGRTGDAGPVGPPGPPGPPGPPGPPSAGFDFSFLPQPPQEKAHDGGRYYRA |
| CF-1552(I) | MDPVVLQRRDWENPGVTQLNRLAAHPPFASDPM(GAPGAPGSQGAPGLQ) n GAMGSS, n＝52 |

2.2 *In vitro* analysis of cell proliferation

To assess cell proliferation using an MTT assay, skin keratinocytes and fibroblasts, which are essential for wound healing, were analyzed. HACAT and HSF cell lines were seeded into 96-well plates at a density of 5×10³ cells per well. After allowing the cells to adhere, the original culture medium was replaced, and the wells were rinsed with PBS. The control group was given a serum-free medium, while the experimental group received a serum-free medium containing the samples. Following a 48-h incubation, 50 μL of MTT solution (5 mg/mL) was added to each well. After 2 h, the MTT solution was removed, and 150 μL of DMSO was added to each well. Absorbance at 490 nm was then measured using a Bio-Tek microplate reader. Each concentration was tested in quintuplicate for analysis.

2.3. *In vitro* study of cell migration

To investigate how collagen affects the migration of HACAT and HSF cells, an in vitro wound healing model was set up. HACAT and HSF cells (1×10^6^ cells/well) were seeded into 6-well plates and cultured until a confluent monolayer formed. A wound was created by scratching the monolayer with a sterile 200 μL pipette tip, followed by rinsing with PBS to remove cell debris. The samples were then treated with collagen-containing medium (1 mg/mL) for further incubation. Images of the scratches were captured using a microscope, and the healing process was analyzed with ImageJ (x64) at 0 and 24 h.

2.4. Real-time qPCR

HACAT and HSF cells were cultured in a collagen-containing medium (1 mg/mL) to study its effects on cell differentiation, with the medium being replaced every two days for a total of four changes. After one week, cells were collected into separate centrifuge tubes for RNA extraction. They were lysed with Trizol reagent and incubated at room temperature for 8 min. The lysate was transferred to a new 2 mL microtube, incubated at 25 °C for 10 min, mixed with 200 µL of chloroform, and incubated again at 25 °C for 10 min. Following this, the mixture was centrifuged at 12,000 rpm for 20 min at 4 °C. The supernatant was transferred to a new 1.5 mL microtube, mixed with isopropanol, and incubated at 25 °C for 10 min. After centrifugation at 12,000 rpm for 10 min at 4 °C, the supernatant was discarded, and the pellet was washed with 75% ethanol and centrifuged. The final pellet was dissolved in enzyme-free water. Complementary DNA (cDNA) synthesis was carried out using the RevertAid First Strand cDNA Synthesis Kit (Thermo Fisher Scientific, USA), and quantitative reverse transcriptase-PCR (RT-qPCR) was performed using a Bio-Rad system (USA). β-actin gene RNA expression served as a reference to normalize the RNA expression levels of other genes.

Table 2. Gene primers used in qPCR

| Gene | Primer(5′-3′) | Primer Direction |
| --- | --- | --- |
| Col α(I) | 5'-ACATGCCGTGACCTCAAGAT-3' | Forward |
|  | 5'-ATGTCCATTCCGAATTCCTG-3' | Reverse |
| TGFβ-1 | 5'-ATGGAGAGAGGACTGCGGAT-3' | Forward |
|  | 5'-TAGTGTTCCCCACTGGTCCC-3' | Reverse |
| VIM | 5'-TCCGCACATTCGAGCAAAGA-3' | Forward |
|  | 5'-TGATTCAAGTCTCAGCGGGC-3' | Reverse |

2.5. Circular dichroism spectrum of collagen

To ensure proper calibration of the instrument, including zero calibration and wavelength accuracy, dilute the collagen samples to the appropriate concentration in a solution. First, measure the CD signal of the buffer alone, then measure the CD signal of the sample in the range of approximately 190-250 nm.

2.6. Preparation of HACAT and HSF cell photo aging model

Skin photo-aging primarily affects skin keratin-forming cells, so experiments were carried out using HACAT and HSF cells. These cells were cultured in dishes until they reached approximately 90% confluence, then seeded into 96-well plates at a density of 5000 cells per well. The wells were divided into control and UVB model groups. After 24 h of incubation, the culture medium was removed, the wells were washed with PBS, and each well was filled with 100 μL of PBS. The control wells, which did not require UVB irradiation, were covered with tin foil, while the UVB model wells were exposed to UVB irradiation for 4 h at 15 mJ/cm² using a UVB lamp.

2.7 *In vitro* effect of collagen on the proliferation of photo aging cells

The same modeling procedure described in section 2.5 was applied using a collagen concentration of 1 mg/mL and UVB-type UV lamps with an irradiation intensity of 15 mJ/cm². After 24 h of incubation, 20 μL of MTT solution (5 mg/mL in sterile PBS) was added, followed by the addition of 150 μL of DMSO after 4 h. The cells were then shaken for 10 min on a shaker. The absorbance at 490 nm was measured using an enzyme marker, and the survival rate for each group was calculated.

$Survival rate \left( \% \right)=\left( \frac{A_{model group}}{A_{control group}} \right)\times100\%$ (1)

2.8. *In vitro* effect of collagen on the level of superoxide dismutase (SOD) in photo-aged cells

HACAT and HSF cells were cultured in dishes until reaching approximately 90% confluence. The cells were then transferred to 6-well plates at a density of 1×10^6^ cells per well, grouped, and treated according to the method described in section "2.5". To disrupt the cells and release intracellular components, they were fragmented and centrifuged at 2000-3000 rpm for about 20 min. The supernatant was carefully collected, and the Human Superoxide Dismutase (SOD) enzyme-linked immunoassay (ELISA) kit was used to test the sample. The procedure was followed according to the standard protocol, the absorbance at 450 nm was measured, and the SOD value was determined by comparing the results to a standard curve.

2.9. *In vitro* effect of collagen on the levels of reactive oxygen species (ROS) in photo aged cells

HACAT and HSF cells were cultured in a dish until they reached approximately 90% confluence. They were then transferred to a 6-well plate at a density of 1×10^6^ cells per well, grouped, modeled, and treated according to the protocol outlined in section "2.5" for 24 h. After removing the culture medium, 1 mL of DCFH-DA (10 μmol/L), diluted 1:1,000 in serum-free culture medium, was added. The cells were incubated at 37℃ with 5% CO_2_ for 20 min, with the solution being gently mixed every 5 min to ensure thorough contact between the probe and the cells. The cells were then washed three times with serum-free culture medium followed by three washes with PBS. Finally, 1 mL of PBS was added, and the level of reactive oxygen species damage was assessed using fluorescence microscopy for imaging.

2.10. Wound healing rate

All animal experiments were reviewed and approved by the Northwest University Laboratory Animal Management and Ethics Committee (approval number NWU-AWC-20220904M), ensuring compliance with legal and institutional animal ethics guidelines. Sixty female Kunming mice, each weighing 20±2 g, were used to investigate the effects of collagen on wound healing. The mice were housed in laboratory facilities under a 12h light/12h dark cycle for 7 days to acclimate, with free access to food pellets and drinking water throughout the experiment. After shaving the dorsal hair of the mice, they were randomly divided into 6 groups of 10 mice each. Mice were anesthetized with an injectable 3% pentobarbital sodium solution at a dose of 1.0 mL/kg. A circular wound with an 8 mm diameter was created on the dorsal region, and a collagen solution (1 mg/mL) was applied to the wound using a pipette gun and evenly distributed with a cotton swab once daily. On days 0, 4, 8, and 13, optical images of the wounds were captured using a digital camera from five mice in each group to monitor healing progress, and wound areas were measured accurately using Image J software.

2.11. Histological examinations & Masson staining

Skin samples from three mice were collected at 4, 8, and 13 days postoperatively, fixed in 10% formaldehyde for over 24 h, embedded in paraffin wax, and sectioned into 5 μm slices. These slices were then subjected to hematoxylin and eosin (H&E) and Masson staining, and collagen density was analyzed using ImageJ.

2.12. Immunofluorescence staining

The skin tissue sample, fixed with formaldehyde, was embedded in paraffin wax, cut, and mounted on a glass slide. Immunofluorescence staining was conducted using α-smooth muscle (α-SMA, Servicebio, GB111364), collagen I (Col-I, Servicebio, GB11022-3), and collagen III (Col-III, Servicebio, GB111269). The fluorescence intensity was then observed and analyzed using ImageJ.

2.13. Animal model for photo-aging skin repair

Forty female Kunming mice (weighing 20 ± 2 g) were used to investigate the efficacy of collagen in repairing skin photo-aging. The mice were acclimatized for 7 days in a laboratory with a 12h light/12 h dark cycle, having free access to food and water. Their back hair was shaved (3 cm × 3 cm), and they were then randomly assigned to four groups of 10 mice each. UVA and UVB lamps were positioned 15 cm from the mice's skin to irradiate the exposed areas. The minimum erythema dose (MED) was measured using a UV energy detector, with UVA set at 1.71 J/cm² and UVB at 0.39 J/cm² for a 30-min irradiation. To create a UV-induced photo-aging model, the mice underwent two stages of UV irradiation: in the first phase, they received 1 MED every other day for the initial five weeks, and in the second phase, the dose was increased to 2 MED every other day for the following five weeks. The total UVA dose amounted to 92.34 J/cm², and the total UVB dose was 21.06 J/cm² over the 10-week period. Mice in the treatment group had collagen solution applied to their backs daily, those in the normal-blank group received no UV irradiation or treatment, mice in the untreated-collagen group were treated with collagen but not irradiated, and those in the model group were irradiated without treatment. If any mice developed ulcers or blisters, irradiation was paused 1-2 times until the symptoms resolved, after which the experiment resumed.

2.14. Effect of collagen on malondialdehyde (MDA) levels in the skin of photo damaged mice

At the end of the tenth week, skin tissue was harvested from the backs of the mice following the procedures outlined in "2.12," then cut and weighed. After adding an appropriate amount of PBS, the specimens were homogenized thoroughly. The homogenate was centrifuged at 2000-3000 rpm for approximately 20 min, and the supernatant was carefully collected. Using the human malondialdehyde (MDA) enzyme immunoassay (ELISA) kit, the sample was processed according to the standard protocol. The absorbance at 450 nm was measured with an enzyme marker, and the MDA value of the sample was determined by comparing this measurement with the standard curve.

2.15. Effect of collagen on hydroxyproline (HYP) levels in the skin of photo-damaged mice

At the end of the tenth week following the grouping, modeling, and treatment methods described in "2.12," skin tissue from the back of the mice was collected, cut, and weighed. Approximately 0.2 g of the sample was placed in a glass tube, cut into small pieces to facilitate digestion, and the cap was left slightly loose. To the tube, 2 mL of extract was added, and the mixture was digested by boiling or in an oven at 110℃ for 2 to 6 h, or until no large clumps remained. After digestion, the mixture was cooled, and the pH was adjusted to 6-8 using 10 mol/L NaOH (about 1 mL), ensuring not to over-acidify or over-base. Distilled water was added to bring the total volume to 4 mL. The mixture was then centrifuged at 16,000 rpm for 20 min at 25℃. If impurities remained after centrifugation, they were removed by filtration, and the supernatant was collected for measurement. Black material that might appear during digestion is likely carbonized and does not affect the experiment. The absorbance of the enzyme standard was measured at 560 nm and used to calculate the HYP content using a standard curve.

2.16. Histological examinations & Masson staining

The mice were grouped, modeled, and treated as described in "2.12." At the end of the tenth week, skin tissue samples were collected from their backs and fixed in 10% formaldehyde for over 24 h. These samples were then embedded in paraffin wax, sectioned into 5 μm slices, and stained with hematoxylin and eosin (H&E) and Masson. Collagen density was assessed using ImageJ.

2.17. Immunofluorescence staining

Skin tissue samples were fixed with formaldehyde, embedded in paraffin, and then sectioned and mounted on slides. To detect Type I procollagen and MMP-1, mouse anti-human procollagen I and rabbit anti-human MMP-1 antibodies (FITC, 1:2000 dilution) were used. The fluorescence intensity was observed and analyzed using ImageJ.

2.18. Statistical analyses

Data were expressed as mean ± standard deviation and analyzed using SPSS (IBM SPSS Statistics 19) software. All experiments were repeated at least three times, and significance analysis included one-sided or bilateral t-tests. (Significance was as follows: Compared to the normal group: **p* < 0.05; ***p* < 0.01; ****p* < 0.001, Compared to the control group: ^#^ *p* < 0.05; ^##^*p* < 0.01; ^##^*p* < 0.001).

# 3. Results and Discussion

3.1 *In vitro* Col Ⅰ and CF-1552(I) promotes cell proliferation, migration and intracellular gene differentiation


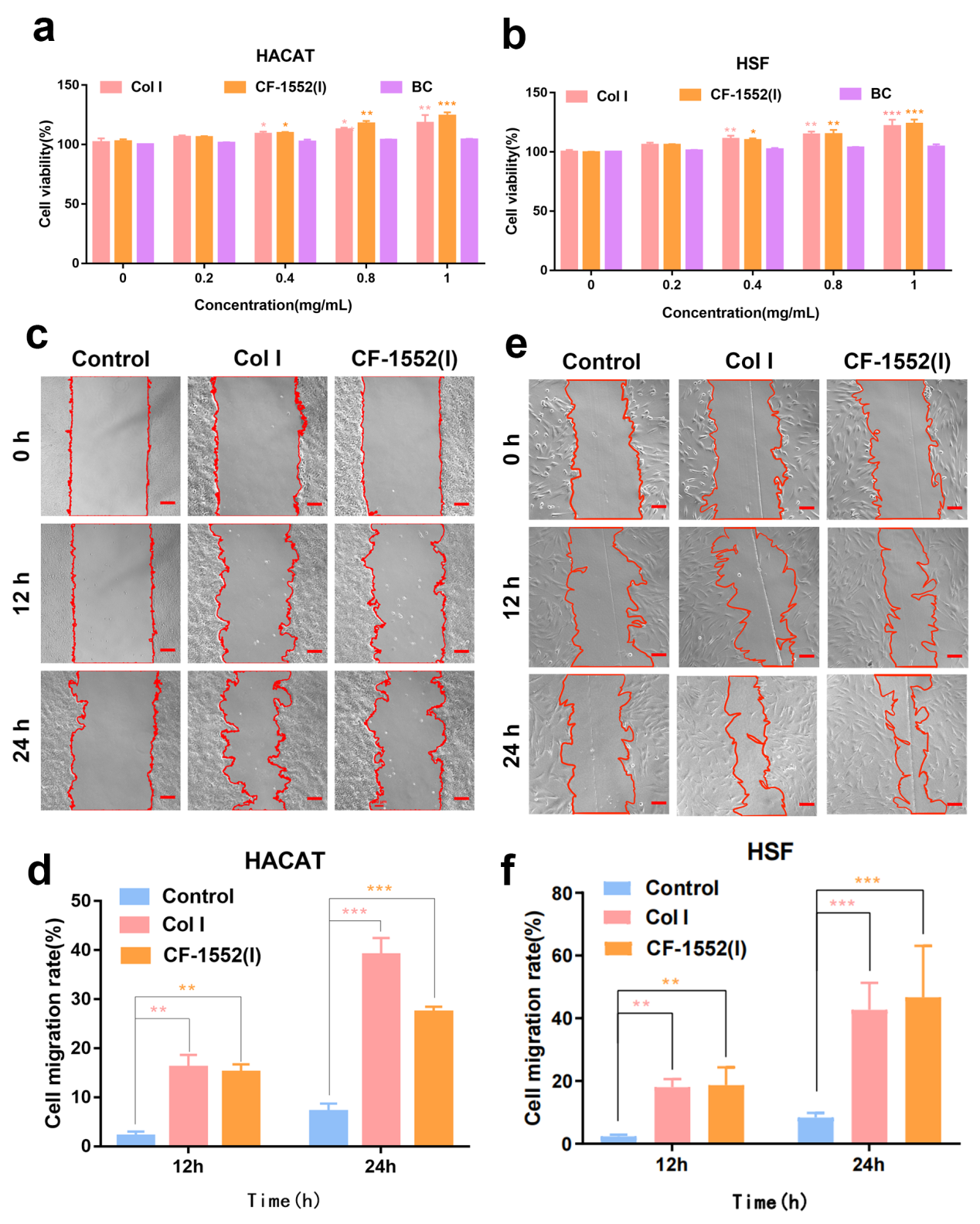


Fig. 1. a) Cell viability (HACAT) with collagen (*p < 0.05, **p < 0.01, n = 3),Specify 100% cell viability for the control group using normal media; b) Cell viability (HSF) with collagen (**p < 0.01, n = 3),Specify 100% cell viability for the control group using normal media; c) Microscope images of *in vitro* scratches for the various experimental groups of HACAT cells. (scale bar: 50 μm); d) Quantified *in vitro* scratch closure results for the various experimental groups of HACAT cells. (**p < 0.01, ***p < 0.001, n = 3); e) Microscope images of *in vitro* scratches for the various experimental groups of HSF cells. (scale bar: 50 μm); f) Quantified *in vitro* scratch closure results for the various experimental groups of HSF cells. (**p < 0.01, ***p < 0.001, n = 3).

To explore how collagen influences the intricate process of wound healing, we conducted in vitro experiments to assess the effects of Col I and CF-1552(I) on cell proliferation, migration, and intracellular gene differentiation.[25-27].

3.1.1. Cell proliferation

Skin wound healing is a complex physiological process, with re-epithelialization being a critical step that involves the migration and proliferation of keratinocytes and skin fibers to cover the exfoliated dermal surface[28]. Therefore, cells were cultured using collagen to observe cell proliferation. HACAT and HSF were treated with serum-free cultures containing different concentrations of Col I, CF-1552(I) and BC collagen (0, 0.2, 0.4, 0.8, and 1 mg/mL) for 48 h to assess cell viability. Figures 1a and 1b illustrate that cell proliferation rates increased with higher collagen concentrations. Specifically, HACAT and HSF cells treated with 1 mg/mL CF-1552(I) showed proliferation rates of 132.22% and 125.37%, respectively, compared to the control group (0 mg/mL), while cells treated with 1 mg/mL Col I had proliferation rates of 127.35% and 123.22%, respectively. In the early stages of wound repair, collagen promotes extensive proliferation of immortalized epidermal cells and fibroblasts. Subsequently, fibroblasts transform into myofibroblasts, aiding in wound contraction[29, 30]. The effectiveness of Col I and CF-1552(I) in enhancing skin healing at the cellular level was partially confirmed by their notable pro-proliferative properties. However, collagen BC from the bovine Achilles tendon did not exhibit significant effects on cell proliferation throughout the process, likely due to its insolubility in the neutral culture medium. Consequently, BC was not used in subsequent experiments for control.

3.1. 2. In vitro wound closure

To study the effect of collagen on cell migration during the complex wound-healing process, we cultured cells in a collagen-containing medium and observed their migration, particularly in scratched areas [31] [32]. After treating HACAT cells with medium containing 1 mg/mL Col I and CF-1552(I) for 24 h, the cell migration rates were 37.5% (P<0.001) and 29.5% (P<0.001), respectively, as shown in Fig. 1C and 1D. Similarly, treatment of HSF cells with the same medium for 24 h resulted in cell migration rates of 42.5% (P<0.001) and 45.5% (P<0.001), as depicted in Fig.1F and 1G. These results indicate that both Col I and CF-1552(I) effectively promote cell migration and accelerate cellular scratch healing, with no significant difference observed between the two treatments in enhancing HSF cell migration.

3.1.3. Intracellular gene differentiation

Collagen type I (Col α(I)), a crucial component of the extracellular matrix (ECM), plays a significant role in wound healing and tissue strength[33]. The fibroblast proliferation essential for this process relies on TGF-β1[34]and the introduction of fibroblasts enhances the synthesis and deposition of Col α(I), thereby increasing the tensile strength of the wound[35-37]. Additionally, myofibroblast contraction drives the wound healing process. Genes and proteins influencing wound healing and differentiation are associated with vimentin (VIM)[38, 39]. To assess the impact of collagen on the promotion of intracellular Col α(I), TGF-β1, and VIM differentiation, RT-qPCR was employed. Cultures with collagen at 1 mg/mL concentration stimulated mRNA levels linked to HACAT and HSF differentiation. Fig. 2a and Fig. 2b illustrate that inHACAT and HSF cells cultured with CF-1552(I), the expression of Col α(I) and TGF-β1 increased significantly by 5.9-fold (p<0.001) and 1.67-fold (p<0.05), respectively. Additionally, waveform protein expression rose by 2.7-fold (p<0.05) compared to the control group. When comparing the effects of Col I and CF-1552(I) on gene differentiation, Col I treatment resulted in a 4.9-fold increase in waveform protein expression inHACAT cells, which was notably higher than the 2.7-fold increase observed with CF-1552(I) (p<0.01). During the later stages of wound healing, the expression of Col α(I) and TGF-β1 proteins was significantly enhanced. Both Col I and CF-1552(I) promoted the differentiation of TGF-β1, leading to increased synthesis and deposition of Col α(I). However, there was no significant difference between Col I and CF-1552(I) in their ability to enhance TGF-β1 expression. Vimentin, which supports cytoskeletal stability, was also upregulated by both Col I and CF-1552(I), indicating their role in promoting cellular stability.

3.1.3. Triple helix structure of Col I and CF-1552(I) materials

In general, natural collagen is a typical triple-stranded superhelical structure assembled from three left α-helices twisted together, and its CD is usually characterized by displaying a significant negative absorption peak near 198 nm and a smaller positive absorption peak near 220 nm, and these two absorption peaks demonstrate the triple-helical structure of Col I [40-42] (Fig.2d). However, during the denaturation process of collagen, the absorption peak at 220 nm will decrease as the degree of denaturation deepens, while the absorption peak at 198 nm tends to be significant. As shown in the Fig.2c, CF-1552(I) protein has a very obvious negative absorption peak at 198 nm, but a small and broad positive absorption peak at 220 nm, which may be due to the existence of an irregular coil structure in the secondary structure of the protein [43]. The spectral features of CF-1552(I) are similar to those of denatured collagen [44]，which suggests that the CF-1552(I) protein is collagen that has not formed a three-stranded helical structure.


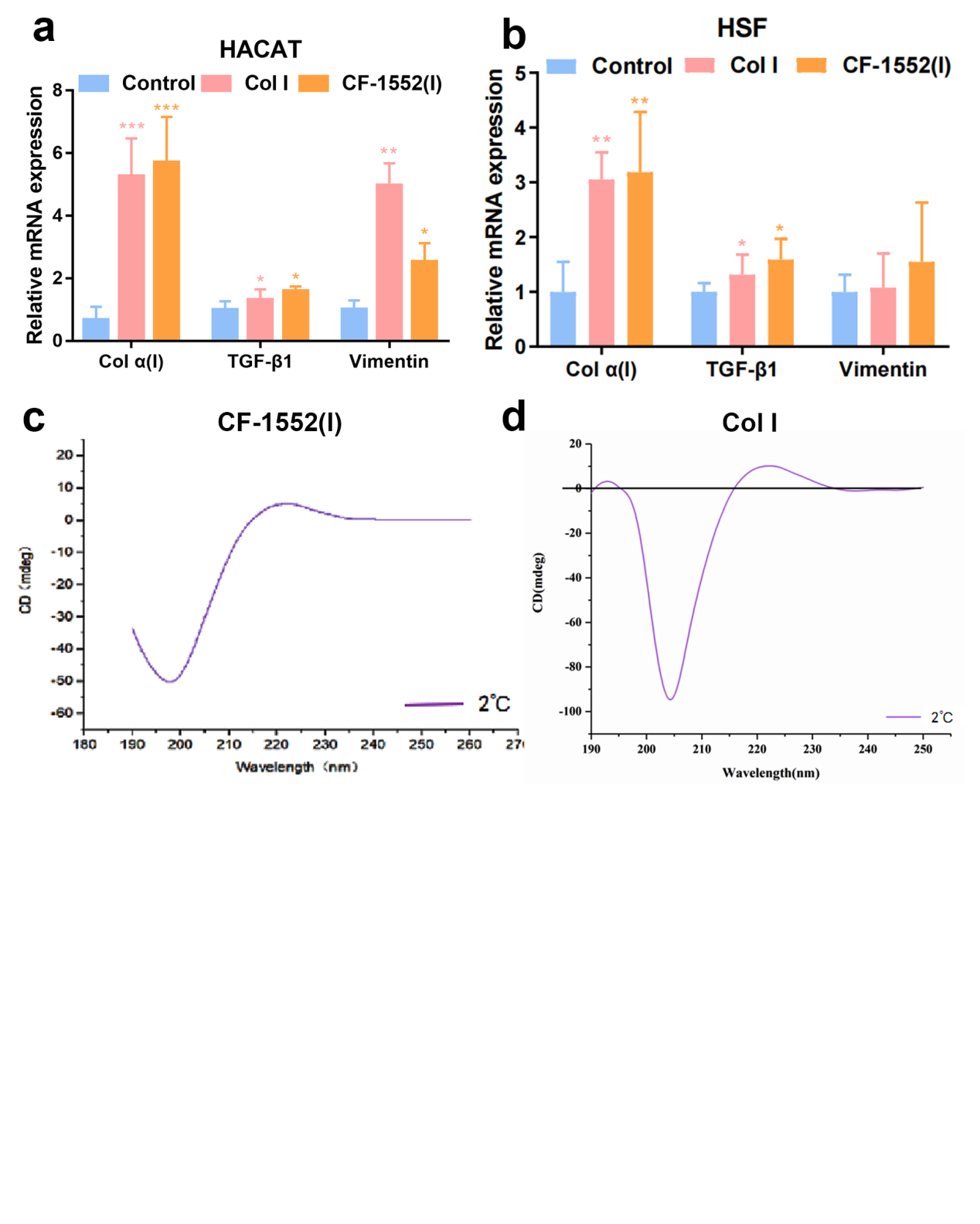


**Fig. 2.** a) Differentiation of promoted genes Col α(I), TGF-β1 and Vimentin in cells (HACAT) treated with different collagen.( **p* < 0.05, ***p* < 0.01, ****p* < 0.001, n = 3); b) Differentiation of promoted genes Col I α(I), TGF-β1 and VIM in cells (HSF) treated with different collagen. (**p* < 0.05, ***p* < 0.01, n = 3); c) circular dichroism of the CF-1552(I) materials; d) circular dichroism of the Col I materials.

3.2 Effect of *in vitro* Col I and CF-1552(I) on cellular level photo-aging repair

UVB and, to a lesser extent, UVA irradiation cause damage to human skin, which leads to sun-induced skin diseases. UVB exposure initiates photo-oxidation reactions that disrupt antioxidant defenses and elevate cellular reactive oxygen species (ROS) levels. The extent of ROS-induced damage is moderated by antioxidants like superoxide dismutase (SOD), which helps maintain physiological balance[45, 46]. Therefore, we investigated the effects of Col I and CF-1552(I) on intracellular ROS and SOD levels under UVB irradiation by *in vitro* experiments.


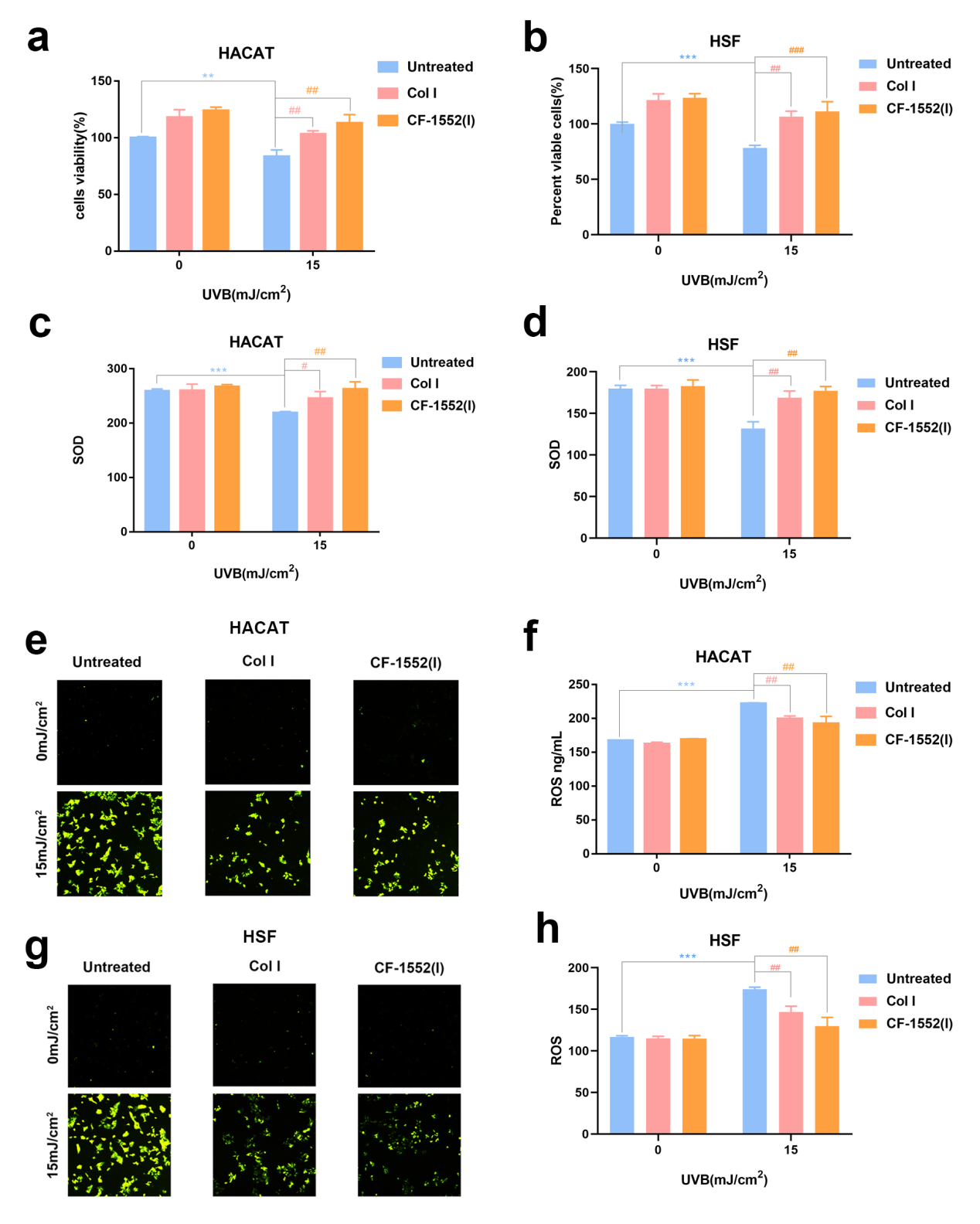


**Fig. 3.** a) Detection of changes in viability of cells(HACAT) treated with different collagen after irradiation (UVB = 15 mJ/cm^2^) compared to cells not irradiated. (UVB = 0 mJ/cm^2^) (Compared to the normal group: ***p* < 0.01, Compared to the control group: ##*p* < 0.01, n = 3); b) Detection of changes in viability of cells(HSF) treated with different collagen after irradiation (UVB = 15 mJ/cm^2^) compared to cells not irradiated. (UVB = 0 mJ/cm^2^) (Compared to the normal group: ***p* < 0.01, Compared to the control group: ##*p* < 0.01, n = 3); c) Changes in the content of SOD in cells (HACAT) treated with different collagen after irradiation (UVB=15mJ/cm^2^) were detected by the kit. (Compared to the normal group: ****p* < 0.001, Compared to the control group: #*p* < 0.05, ##*p* < 0.01, n = 3); d) Changes in the content of SOD in cells (HSF) treated with different collagen after irradiation (UVB=15mJ/cm^2^) were detected by the kit. (Compared to the normal group: ****p* < 0.001, Compared to the control group: #*p* < 0.05, ##*p* < 0.01, n = 3); e) ROS fluorescent probe (DCFH) labeled cells (HACAT) after irradiation (UVB=15mJ/cm^2^) to detect intracellular reactive oxygen species damage. The fluorescence indicates the intracellular ROS content; f) Intracellular (HACAT) ROS levels after collagen treatment were measured with the ROS kit. (Compared to the normal group: ****p* < 0.001, Compared to the control group: ##*p* < 0.01, n = 3); g) ROS fluorescent probe (DCFH) labeled cells (HSF) after irradiation (UVB=15mJ/cm^2^) to detect intracellular reactive oxygen species damage. The fluorescence indicates the intracellular ROS content; h) Intracellular (HSF) ROS levels after collagen treatment were measured with the ROS kit. (Compared to the normal group: ****p* < 0.001, Compared to the control group: ##*p* < 0.01, n = 3)

3.2.1. Col I and CF-1552(I) repairs the cytotoxicity of UVB irradiation in HACAT and HSF cells

We investigated the impact of collagen on the cytotoxicity of UVB irradiation by assessing cell viability[47, 48]. As depicted in Fig. 3a, the viability of UVB-irradiated HACAT cells (15 mJ/cm²) dropped to 83.39±5.91%, a significant decrease compared to non-irradiated cells (0 mJ/cm²) (**p < 0.01). However, after treatment with Col I, the cell viability increased to 103.21±2.98%, and treatment with CF-1552(I) raised it to 113.07±7.40%, showing a significant improvement compared to the untreated group (##p < 0.01). Similarly, Fig. 3b illustrates that UVB-irradiated HSF cells (15 mJ/cm²) had a viability of 78.41±2.31%, significantly lower than that of non-irradiated cells (**p < 0.01). After Col I treatment, viability increased to 106.74±4.86%, and CF-1552(I) treatment led to 111.36±8.75%, both showing significant enhancements over the untreated cells (##p < 0.01). At the cellular level, collagen was found to improve the viability of both HACAT and HSF cells post-UVB irradiation, with no significant differences between the Col I and CF-1552(I) treatments. Collagen exhibited a proliferative effect on both cell types, accelerating cell renewal and enhancing overall viability despite UV-induced damage. Thus, both Col I and CF-1552(I) effectively restored cell viability after UVB irradiation.

3.2.2. Effect of Col I and CF-1552(I) on the amount of SOD in HACAT and HSF cells after UVB irradiation.

As shown in Fig. 3c, UVB irradiation (15 mJ/cm²) significantly decreased the intracellular SOD content in HACAT cells compared to non-irradiated cells (0 mJ/cm²) (***p < 0.001). When these UVB-irradiated HACAT cells were cultured with a medium containing Col I and CF-1552(I), the intracellular SOD content increased significantly. Notably, cells cultured with CF-1552(I) showed a more pronounced increase in SOD content compared to those cultured with Col I (#p < 0.05, ##p < 0.01). Similarly, Fig. 3d illustrates that UVB irradiation (15 mJ/cm²) reduced the intracellular SOD content in HSF cells compared to the non-irradiated controls (0 mJ/cm²) (***p < 0.001). UVB-irradiated HSF cells cultured with Col I and CF-1552(I) also exhibited a significant increase in SOD content. Again, cells treated with CF-1552(I) demonstrated a more significant rise in SOD content than those treated with Col I (##p < 0.01). Overall, both Col I and CF-1552(I) were effective in increasing SOD expression, with no significant difference between the two treatments in elevating SOD levels in the cells.

3.2.3. Effect of Col I and CF-1552(I) on the amount of intracellular ROS in HACAT and HSF cells after UVB irradiation.

Fig. 3e and 3f demonstrate that HACAT cells exposed to UVB irradiation (15 mJ/cm²) exhibit a significant increase in intracellular ROS levels compared to non-irradiated HACAT cells (0 mJ/cm²) (***p < 0.001). When these UVB-irradiated cells were cultured in media containing Col I or CF-1552(I), there was a marked reduction in ROS levels compared to the untreated group (##p < 0.01). This suggests that both Col I and CF-1552(I) have a cellular-level reparative effect on photo-aging. Comparison between the Col I and CF-1552(I) treatments indicated no significant difference in their efficacy in reducing ROS levels, implying that both treatments are equally effective in mitigating UVB-induced cellular damage.

Similarly, Fig.3g and 3h show that HSF cells subjected to UVB (15 mJ/cm²) also demonstrated a significant increase in intracellular ROS compared to those without UVB exposure (0 mJ/cm²) (***p < 0.001). Culturing UVB-irradiated HSF cells in media with Col I or CF-1552(I) resulted in a significant reduction in ROS levels compared to the untreated group (##p < 0.01), reinforcing the reparative effect of Col I and CF-1552(I) on photo-aging at the cellular level. No significant difference was observed between the two treatments in their impact on ROS reduction, further indicating their equivalent effectiveness in repairing UVB-induced ROS damage.

UVB radiation leads to the excessive production of superoxide anions in both HACAT and HSF cells, disrupting the cellular oxidative balance. The cell's endogenous antioxidant defenses are insufficient to counteract this ROS overproduction, necessitating an increase in intracellular antioxidant enzyme expression. The SOD assay results show that both Col I and CF-1552(I) enhance intracellular SOD expression and reduce ROS levels. Consequently, Col I and CF-1552(I) are effective in increasing antioxidant enzyme levels and protecting HACAT and HSF cells from oxidative stress induced by UVB radiation. Moreover, there is no significant difference between the two treatments regarding their effectiveness in reducing ROS content.

3.3 Skin wound repair: mouse dorsal skin defect wound model

To assess the effectiveness of Col I and CF-1552(I) in wound repair, we conducted experiments on common wounds on the backs of mice. This included analyzing the wound area, collagen deposition, and gene expression during the healing process to evaluate how collagen influences skin wound repair [49, 50].

3.3.1. Wound healing effect of the Col I and CF-1552(I).

Skin injuries on the backs of mice were used to model ordinary wounds[51], as depicted in Fig. 4a and Fig. 4b, with the results of several parallel experiments analyzed in Fig. 4c. On day 4, the Col I group exhibited better wound contractility compared to the control group (*p < 0.05). By day 8, the Col I group's advantage over the control group was more pronounced, and the CF-1552(I) group also demonstrated significant wound contractility. By day 13, wounds in the control group had not fully healed, whereas those in the Col I and CF-1552(I) groups were completely closed (**p < 0.01). This indicates that both Col I and CF-1552(I) were effective in skin wound repair. Notably, the Col I group showed earlier wound repair compared to CF-1552(I), likely because the gene fragment in Col I that promotes early wound repair was absent in CF-1552(I). However, both groups achieved complete wound healing simultaneously, suggesting that the genes in Col I promoting early repair do not influence the final repair outcome.


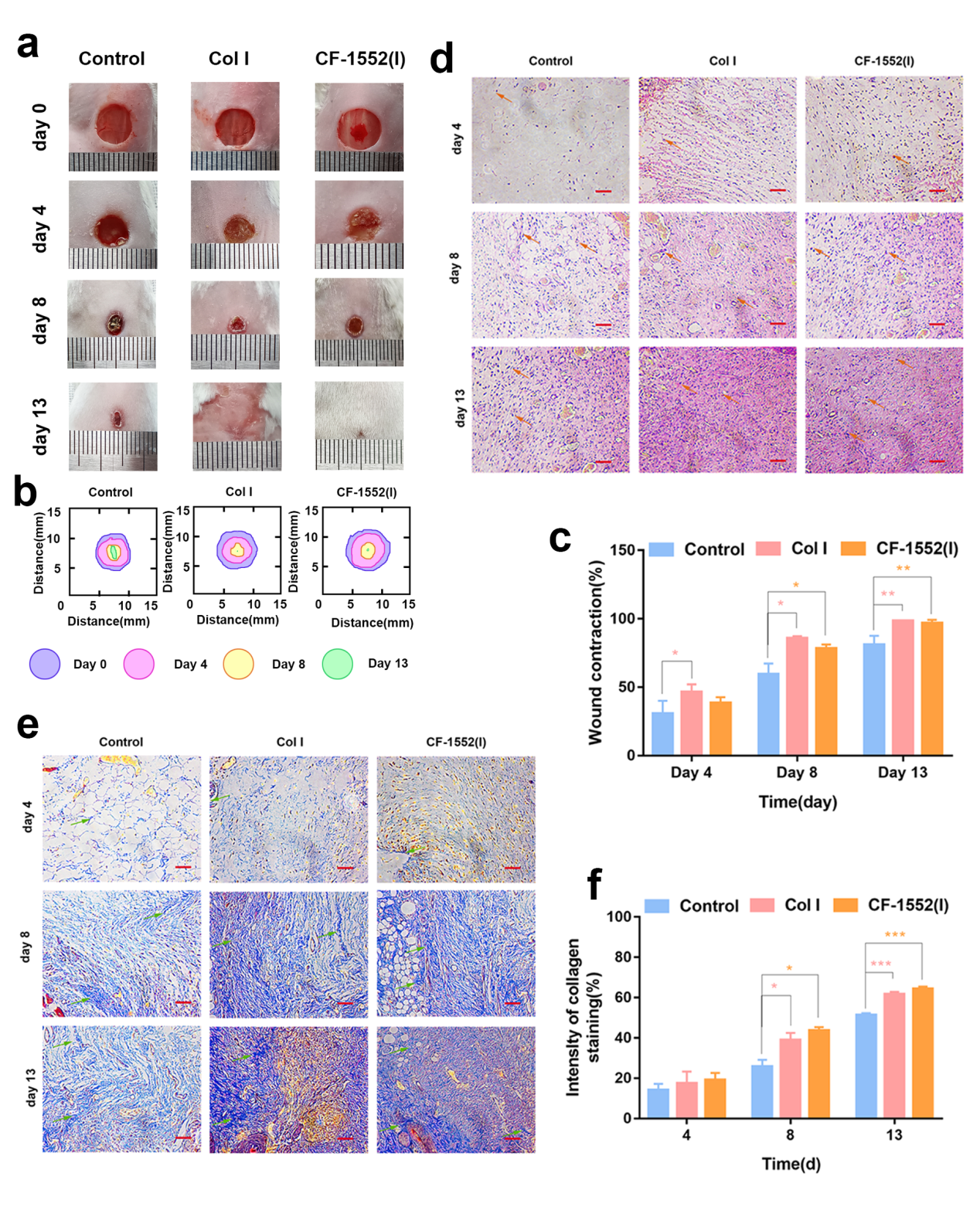


**Fig. 4.** a) Photographs of wounds at days 0, 4, 8, and 13; b) Traces of wound-bed closure on days 0, 4, 8 and 13 for each group; c) Wound contraction for each group. (**p* < 0.05, ***p* < 0.01, n = 3); d) H&E staining of wounds after 4, 8 and 13 days. (blood vessels: orange arrows, scale bar: 100 μm); e) Masson’s trichrome staining of a wound site after 4, 8 and 13 days (Collagen fibers: green arrows, scale bar: 100 μm); f) Analysis of collagen staining intensity. (**p* < 0.05, ****p*< 0.001, n = 3)

3.3.2. Histological evaluation of wound repair

Collagen deposition at the wound site is a key indicator of wound healing[52]. To assess this, Masson staining was utilized on mouse skin samples collected on days 4, 8, and 13 to visualize new collagen formation. As illustrated in Fig. 4e and Fig. 4f, the results revealed significantly higher levels of de novo collagen deposition in the Col I and CF-1552(I) groups compared to the control group on both day 8 and day 13. There was no significant difference between the Col I and CF-1552(I) groups (*p < 0.05, ***p < 0.001), suggesting that both treatments were equally effective in promoting collagen deposition and accelerating wound healing.

Granulation tissue abundance is a key indicator of wound healing, and H&E staining was used to assess its presence in the skin of mice on days 4, 8, and 13. As shown in Fig. 4d, both the Col I and CF-1552(I) groups exhibited fewer inflammatory cells by day 8 and greater angiogenesis by day 13 compared to the control group. The effects of Col I and CF-1552(I) on reducing inflammation and promoting angiogenesis were similar. Collagen, known to enhance VEGF gene expression[53], influences various wound healing processes such as angiogenesis, re-epithelialization, and collagen synthesis[54]. Consequently, both Col I and CF-1552(I) effectively promoted angiogenesis and collagen deposition, with no significant difference between the two in terms of their efficacy.

3.3.3. α-SMA, Col-I and Col-III expression in wound regeneration with various treatment

To further investigate the biological mechanism of collagen in skin wound healing, we quantitatively analyzed the expression of α-SMA, collagen I (Col-I), and collagen III (Col-III)[55, 56]. As illustrated in Fig. 5a and Fig. 5b, both the Col I and CF-1552(I) groups significantly increased α-SMA protein expression compared to the control group on days 8 and 13 (*p < 0.05). Similarly, Fig. 5a and Fig. 5c show that both Col I and CF-1552(I) groups notably enhanced Col-I protein expression on these days (*p < 0.05, **p < 0.01). Furthermore, Fig. 5a and Fig. 5d indicate that on day 13, Col I and CF-1552(I) groups significantly raised Col-III protein levels compared to the control group (*p < 0.05). Both Col I and CF-1552(I) groups had comparable effects in promoting α-SMA, Col-I, and Col-III protein expressions, which suggests that they enhance wound healing by upregulating the TGF-β1 gene and increasing these proteins. Thus, these findings demonstrate that Col I and CF-1552(I) significantly accelerate skin wound repair, with no substantial difference observed between the two treatments in their effects on α-SMA, Col-I, and Col-III protein expression during the healing process.


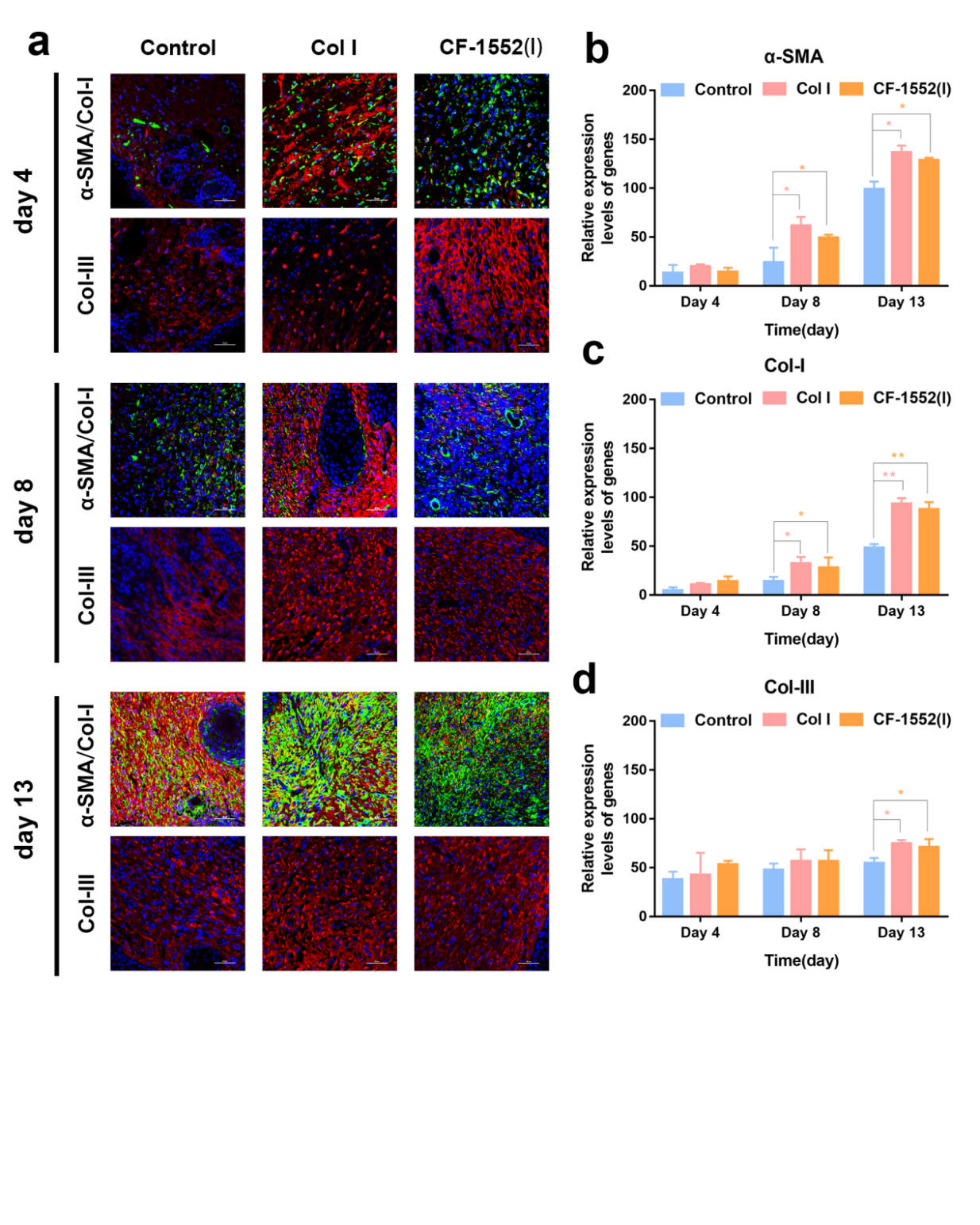


**Fig. 5.** a) Images of immunofluorescence labeling of skin wound tissues with α-SMA (green) , Col-I (red) and Col-III (red). (scale bar: 100 μm); b) α-SMA quantitative analysis of immunofluorescence images. (n = 3); c) Col-I quantitative analysis of immunofluorescence images. (**p* < 0.05, n = 3); d) Col-III quantitative analysis of immunofluorescence images. (**p* < 0.05, ***p* < 0.01, n = 3)

3.4 Skin photo-aging repair: UVB irradiation photo-aging model of mouse dorsal skin


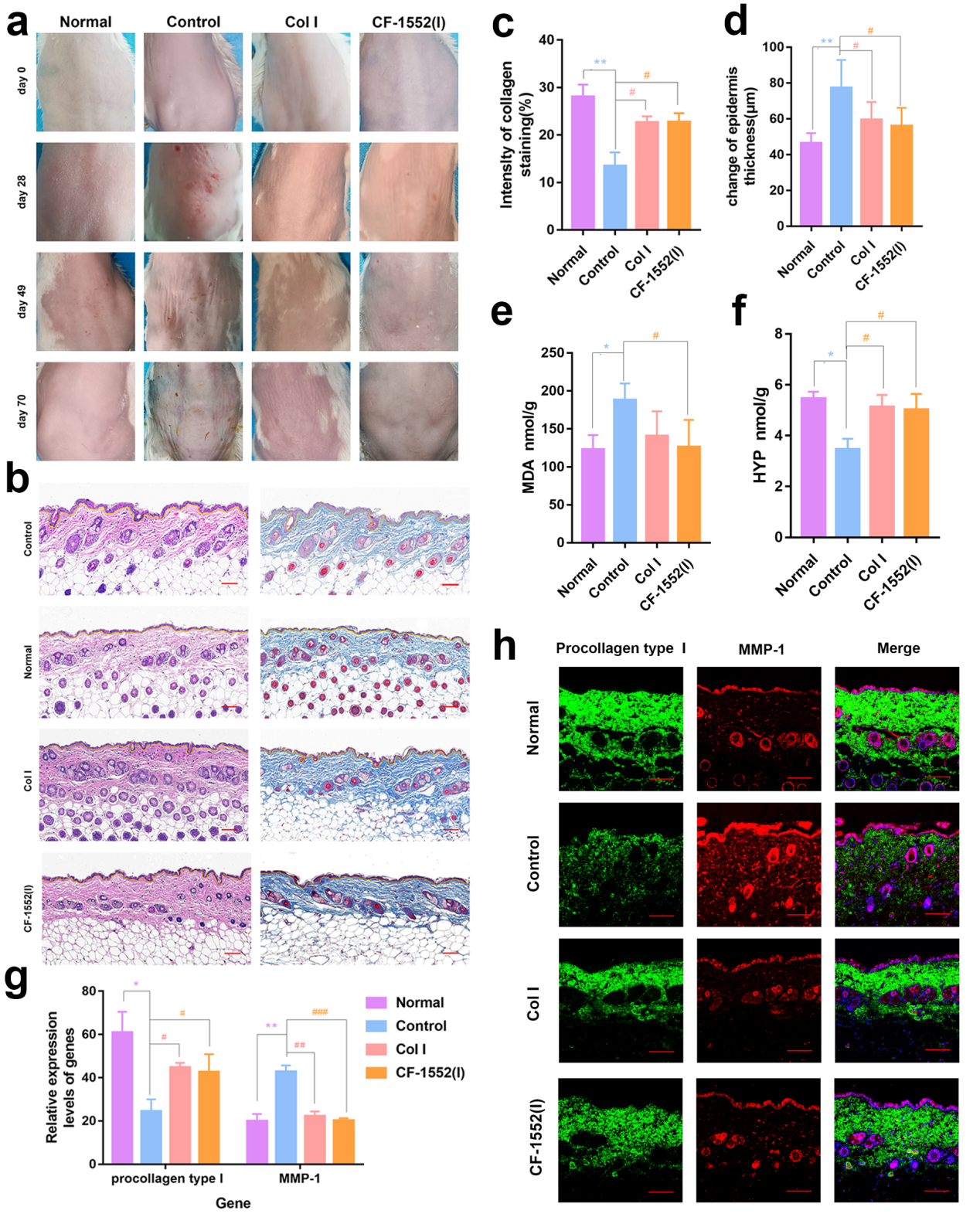


**Fig. 6.** a) The appearance of folds in the dorsal skin of UVB-irradiated mice at days 0,28,49,70. (UVB = 0.39 J/cm^2^); b) H&E and Masson's tricolor staining of the back skin after 70 days. (dermis: yellow lines, scale bar: 100 μm); c) Analysis of collagen staining intensity. (Compared to the normal group: ***p* < 0.01, Compared to the control group: #*p* < 0.05, n = 3); d) Statistical chart of epidermal thickness. (Compared to the normal group: ***p* < 0.01, Compared to the control group: #*p* < 0.05, n = 3); e) Detection of MDA in skin tissue with the kit. (Compared to the normal group: **p* < 0.05, Compared to the control group: #p < 0.05, n = 3); f) Detection of HYP in skin tissue with the kit. (Compared to the normal group: ***p* < 0.01, Compared to the control group: #*p* < 0.05, n = 3); g) Day 70 quantitative analysis of immunofluorescence images; (Compared to the normal group: **p* < 0.05, ***p* < 0.01, Compared to the control group: #*p* < 0.05, ##*p* < 0.01, ###*p* < 0.001, n = 3) h) Images of immunofluorescence labeling of skin wound tissues with procollagen type I (green) and MMP-1 (red) on day 70. (scale bar: 100 μm)

In order to further investigate the repairing efficacy of Col I and CF-1552(I) on UVB irradiation-induced skin aging, we conducted a collagen repairing experiment on photo-aged skin on the back of mice, as well as the determination of related factors to verify the repairing effect of collagen on UVB-induced skin aging.

3.4.1. Non-invasive method for evaluating changes in skin condition

Non-invasive assessment methods, such as observing skin fold production, minimize damage to the animals' skin[57, 58]. As shown in Fig. 6a, irregular wrinkles on the back skin of mice were clearly evident in the control group compared to the normal group at week 10. This wrinkle formation, characterized by a loss of smoothness, aligns with the description of photo-aging in the literature, confirming the successful establishment of an animal model for skin damage. In contrast, the group treated with Col I and CF-1552(I) showed a reduction in the number of wrinkles on the dorsal skin compared to the control group, indicating that both Col I and CF-1552(I) are effective in mitigating wrinkles caused by UVB irradiation-induced skin aging.

3.4.2. Effect of Col I and CF-1552(I) on MDA and HYP levels

Exposure of skin to UVB radiation increases ROS levels, which leads to reduced SOD activity, disruption of physiological homeostasis, and elevated Malondialdehyde (MDA) levels due to accelerated lipid peroxidation[59-61]. To assess the degree of photo-aging and the efficacy of Col I and CF-1552(I) in skin repair, MDA content in mouse skin tissues was measured. As shown in Fig. 6e, MDA levels in UVB-irradiated skin tissues of the Control group (0.39 J/cm²) were significantly higher (*p < 0.05) compared to the Normal group (0 J/cm²) which was not irradiated. Following collagen coating with either Col I or CF-1552(I) in some Control group mice, MDA levels in the CF-1552(I) group were significantly reduced, almost returning to baseline levels compared to the Control group (#p < 0.05). Although MDA levels also decreased in the Col I group compared to the Control group, the difference was not significant. This suggests that both Col I and CF-1552(I) help mitigate UV-induced ROS increase by enhancing SOD activity, thereby reducing lipid peroxidation and MDA levels. However, CF-1552(I) proved to be more effective than Col I in reducing MDA content, likely due to its rapid degradation into peptides and the increased permeability of active ingredients through the skin. To study the biological activity of CF-1552（I）degradation products, we hydrolyzed CF-1552（I）with pepsin and trypsin, following established research methods[62]. The enzymes were inactivated by heating the mixture in a boiling water bath for 10 min, then centrifuging at 25℃ and 10,000g for 10 min. The supernatant was collected, and the degradation products were isolated using a dialysis bag with a molecular weight cutoff of 100-500 Da (Solarbio, YA1069). SDS-PAGE analysis showed that the degraded CF-1552（I）had a molecular weight of about 10 kDa (Fig. S1a). We tested the effects of 1 mg/mL degradation products on the proliferation of HACAT and HSF cells and found that they significantly enhanced cell proliferation compared to CF-1552（I） (P<0.01) (Fig. S1b).

The antioxidant properties of collagen may stem from its high content of glycine (G), proline (P), and charged amino acid residues, such as arginine (R)[63, 64]. In our study, CF1552 (I), which is rich in G, P, and R, effectively captures and eliminates reactive oxygen species. Therefore, it can be inferred that CF1552 (I) and its degradation products have strong antioxidant properties in comparison to CF1552 (I).

Given CF-1552（I）’s superior MDA reduction effect over Col I, we hypothesized this might be due to its degradation products. Thus, we focused on their antioxidant properties. At 1 mg/mL, the degradation products exhibited higher antioxidant activity than CF-1552（I）, as evidenced by greater DPPH scavenging efficiency (P<0.05) (Fig. S1c). Additionally, the degradation products improved UVB-induced oxidative stress in HACAT and HSF cells, restoring SOD levels more effectively than CF-1552（I）at the same concentration and significantly reducing MDA content (Fig. S1d, S1e). These findings indicate that the antioxidant effect of CF-1552（I）degradation products is comparable to CF-1552（I）itself, aligning with numerous studies reporting enhanced antioxidant and biological activity of collagen peptides post-degradation[65-67]. A more comprehensive study on the degradation products of CF1552 will be conducted in our future research.

Hydroxyproline (HYP), an important indicator of skin aging, is a key amino acid in collagen and serves as a measure of collagen content in the dermis[68]. To assess the extent of photo-aging and the effectiveness of Col I and CF-1552(I) in skin repair, we measured HYP levels in mouse skin tissues. As depicted in Fig. 6f, UVB irradiation (0.39 J/cm²) led to a significant reduction in HYP content compared to non-irradiated controls (0 J/cm²) (**p < 0.01). However, in UVB-irradiated mice, treatment with Col I and CF-1552(I) resulted in increased HYP levels compared to the control group, with both treatments showing positive effects (#p < 0.05). Under normal conditions (UVB = 0 J/cm²), collagen does not affect HYP levels. Overexpression of elastase disrupts collagen and elastin in the extracellular matrix, but Col I and CF-1552(I) enhance HYP expression by downregulating elastase. Our results indicate that both Col I and CF-1552(I) effectively boost HYP content in photo-aged skin, with no significant difference in their enhancement capabilities.

3.4.3. Histological evaluation of the skin

Epidermal thickening, a hallmark of photo-aging, was assessed using H&E staining across various groups of representative skin tissues to evaluate epidermal and dermal thicknesses[69]. As depicted in Fig. 6b and Fig. 6d, the control group exhibited a notable increase in epidermal thickness following UVB (0.39 J/cm²) irradiation compared to the normal group (*p < 0.05, **p < 0.01). Conversely, treatment with Col I and CF-1552(I) resulted in a thinner epidermal layer relative to the control group. Prolonged UVB exposure also led to a reduction in collagen content within the skin, which was measured using Masson staining. As shown in Fig. 6b and Fig. 6c, collagen content in the UVB-irradiated control group significantly decreased compared to the normal group without UVB exposure (0 J/cm²). However, treatment with Col I and CF-1552(I) restored collagen levels to near-normal conditions. The effectiveness of these treatments was consistent across both Col I and CF-1552(I) groups. Increased ROS due to UV irradiation alters dermal connective tissue proteins, contributing to thicker skin and reduced collagen deposition[70]. Col I and CF-1552(I) effectively lower ROS levels, thereby improving skin thickness and collagen content. Additionally, there were no significant differences between Col I and CF-1552(I) regarding their effects on skin thickness recovery and collagen deposition post-UV irradiation.

3.4.4. Expression of procollagen type I and MMP-1 in skin repair under different treatment conditions

Matrix metalloproteinases (MMPs), including MMP-1, are crucial in destructive processes such as inflammation, tumor invasion, and skin aging. Specifically, MMP-1, or mesenchymal collagenase, begins the breakdown of type I, II, and III collagen in the skin[71] [72]. UVB irradiation increases MMP-1 expression in normal human skin while decreasing procollagen type I levels. Our study measured the expression of MMP-1 and procollagen type I under UVB exposure (0.39 J/cm²)[73]. As depicted in Figures 6g and 6h, UVB exposure significantly elevated MMP-1 expression and reduced procollagen type I in the control group compared to the normal group (**p < 0.01 for MMP-1 and *p < 0.05 for procollagen type I). Treatment with Col I and CF-1552(I) led to a decrease in MMP-1 levels and a restoration of procollagen type I expression compared to the control group, with CF-1552(I) showing a more pronounced effect on MMP-1 inhibition (#p < 0.05, ##p < 0.01). Both Col I and CF-1552(I) effectively repaired UV-induced skin damage by inhibiting MMP-1 and restoring procollagen type I. There was no significant difference in the efficacy of Col I and CF-1552(I) in restoring procollagen type I levels after UV irradiation.

.


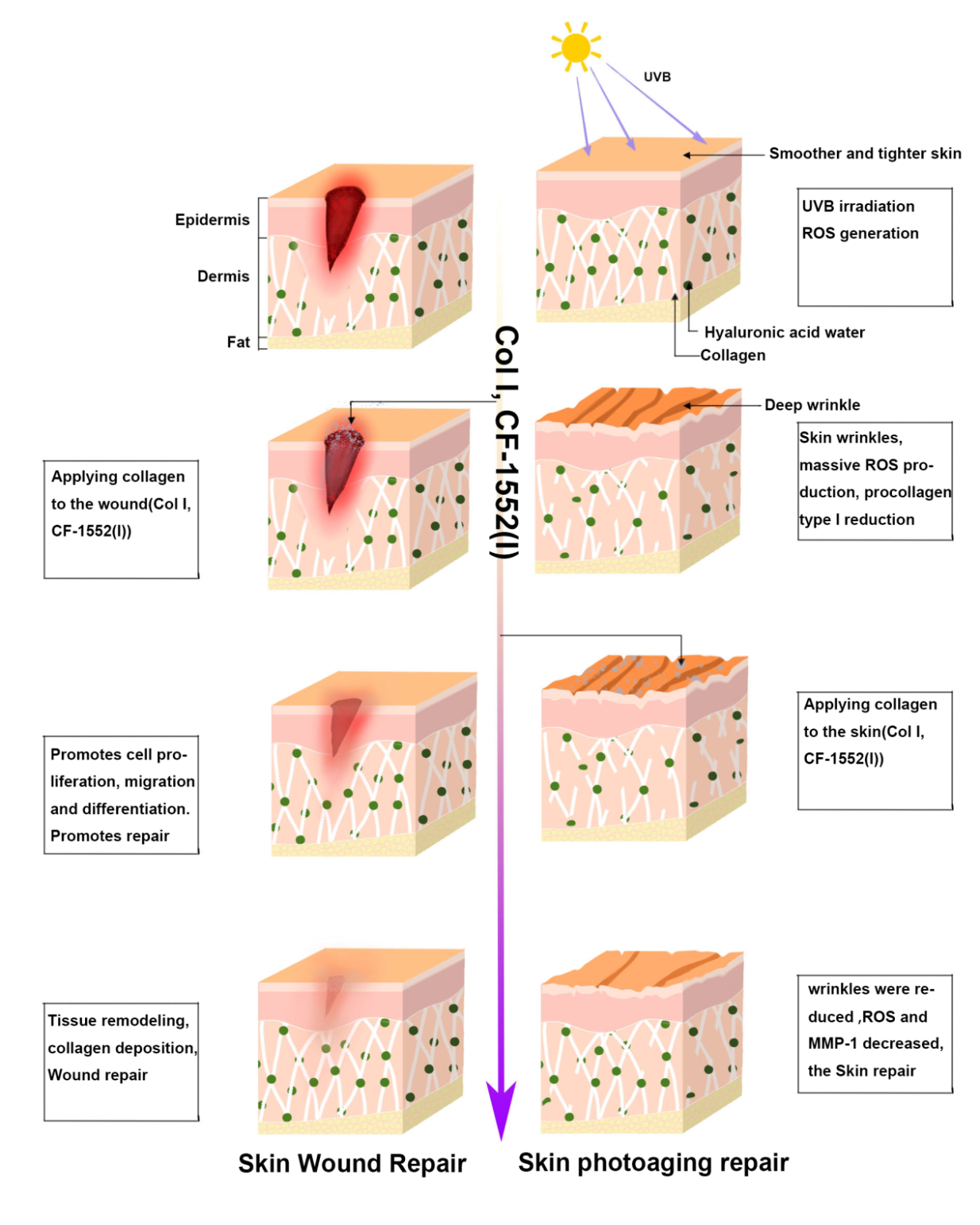


**Fig. 7.** Skin wound repair and skin photo-aging repair mechanisms

# 4. Conclusion

Recombinant collagen, known for its biocompatibility, stability, controlled quality, and low viral risk, was investigated for its effectiveness in skin repair. In the field of skin tissue engineering, a lot of research has been carried out on collagen materials for skin repair and their potential applications. Table S1 describes the studies carried out by various researchers using different sources of collagen as a key ingredient in different formulations (e.g., fish skin collagen, collagen cream, collagen peptide, and collagen hydrogel). Through their study, the authors observed the effective wound healing activity of fish skin collagen from Nile tilapia skin. The results showed that the wound healing rate was 38.8% ± 22.8% in the fish skin collagen treated group and 8.7% ± 17.2% in the fish skin collagen treated group, whereas the in vitro scratch healing rate of fish skin collagen was significantly higher than that of the model control group at a concentration of 50.0 μg/mL. The PSD18 wound healing rate in the control group was 72.1% ± 13.9%, and the best wound healing was achieved with fish skin collagen. In addition, histological assessment showed that fish skin collagen promoted new epidermal layer formation, proliferation, fibroblast formation and new capillarization compared to the model control group[74]. Wounds severely affect the external skin barrier. Further research describes a new approach using bovine collagen cream (BCC) to promote wound healing. In this study, researchers used the NIH 3T3 fibroblast cell line showing increased cell viability without toxicity [75]. Another study reported the role of collagen peptides isolated from jellyfish in wound healing. The results showed that collagen peptide-treated cells exhibited significant scratch closure at 18h, 36h, and 48h at a concentration of 6.25 μg/mL compared to the control group.In in vivo studies, rapid contraction of whole-layer wounds, followed by an increase in collagen deposition and fibroblast growth factor, has been fruitful[76]. In order to accelerate the wound healing process, the authors reported the combination of guanosine tetrahedral hydrogel with recombinant human-derived collagen (G4-RHC). The obtained hydrogel had dermatogenic wound healing properties. In vitro wound healing experiments with L929 cells treated with G4-RHC hydrogel showed that there was rapid migration of L929 cells after G4-RHC hydrogel treatment as compared to the control group. the RHC caused fibroblasts and macrophages to turn over toward the wound, which initiated proliferation and migration [77].Thickening of skin damage and even dermatitis caused by long-term exposure to UV rays is also an urgent problem in the field of skin tissue engineering. Through their research, the authors designed recombinant type III collagen rhCol III with significant antioxidant capacity by synthetic biology technology, and the results of the eighth week of experiments showed that it could reduce skin photoaging caused by UV radiation, including reducing the thickening of the epidermis and dermis The results show that rhCol III can reduce UV-induced photodamage, including reduction of epidermal and dermal thickening, increase of collagen type I (Col I) and collagen type III (Col III) secretion, and extracellular mesenchymal remodeling (ECM) [78].High purity collagen was extracted from porcine skin, the proliferation rate of HACAT and HSF cells treated with porcine skin collagen was not significantly different from that of control cells, and the number of migrating cells was increased by about 40% at a collagen concentration of 0.6 mg/mL compared with the control group, and the proportion of collagen type I and type III fibers was significantly increased after 1 month of injection of porcine skin collagen to delay the aging of the skin [79]In one study, non-denatured type I collagen (YCI) was extracted from yak skin, the relative proliferation rate of human skin fibroblasts (HFF-1) in 0.1 mg/mL YCI solution gradually increased to 120% on day 3, and 150% on day 5, which indicated that YCI significantly promoted the proliferation of HFF-1, and the content of hydroxyproline increased to 106.13% after the treatment of YCI, which indicated that the dermal collagen content increased, and the proportion of migrating cells increased significantly after injection of pig skin collagen for 1 month to delay skin aging[6] . The increase in dermal collagen content and the significant recovery of collagen content to normal levels within 4 days indicated that YCI has the ability to promote dermal collagen regeneration and accelerate the healing of severe UV-mediated skin damage [80].Huang et al. obtained collagen polypeptide (SCSCP) with collagenase inhibitory activity from silver carp (Hypophthalmichthys molitrix) skin by optimizing the enzymatic process, and SCSCP at a concentration of 10 mg/mL possessed high DPPH and hydroxyl radical scavenging activities, whereas 0.1 mg/mL SCSCP significantly restored UVB-induced viability of L929 cells and inhibited UVB irradiation after L929 cell MMP-1 secretion after UVB irradiation to achieve anti-photo-aging effects [81].

Although collagen and its derivatives from a variety of different sources have been used in the field of skin damage repair. However, almost all collagen used for biomedical and pharmaceutical applications and tissue-engineered medical products (TEMP) is primarily of animal origin[82]. However, safety factors should be considered when using animal-derived collagen, including the risk of transmitting viruses and diseases, pathogenicity, and allergic reactions[83].

We preferred to produce the full-length or fragment of the functional amino acid sequence encoded by the human type I collagen gene by synthetic biology techniques, so that the amino acid sequences of Col I and CF-1552 are identical to those of natural collagen, and this purely natural amino acid sequence does not include any recognition site for the discoidal structural domain receptor (DDR), and does not affect matrix metalloproteinases (including membrane type 1 (MT1- MMP), MMP1 and MMP2) expression[84, 85] and is a promising starting material for tissue-engineered medical product matrices.

In this study, both Col I and CF-1552(I) collagen materials were simultaneously evaluated for their efficacy in wound repair and photo-aging skin repair. The results demonstrated that both types of collagen effectively promote skin wound healing and mitigate photo-aging. Compared with Col I, CF-1552(I), as a functionally enhanced small molecule collagen, has shown comparable skin repair performance in some experiments, indicating that CF-1552(I)has a more promising future in skin repair.

Funding

This work was supported by the National Key R&D Program of China (2019YFA0905200).

Supplementary data

Supplementary data are available at Regenerative Biomaterials online.

Conflicts of interest statement

There are no conflicts to declare.

References

1. Ojeh N, Pastar I, Tomic-Canic M, Stojadinovic O. Stem Cells in Skin Regeneration, Wound Healing, and Their Clinical Applications. *Int J Mol Sci* 2015;16:25476-501.

2. Da L-C, Huang Y-Z, Xie H-Q. Progress in development of bioderived materials for dermal wound healing. *Regenerative Biomaterials* 2017;4:325-334.

3. Griffiths CE. Drug treatment of photoaged skin. *Drugs Aging* 1999;14:289-301.

4. Eming SA, Martin P, Tomic-Canic M. Wound repair and regeneration: mechanisms, signaling, and translation. *Sci Transl Med* 2014;6:265sr6.

5. Chen Y, Cai J, Liu D, Liu S, Lei D, Zheng L, Wei Q, Gao M. Zinc-based metal organic framework with antibacterial and anti-inflammatory properties for promoting wound healing. *Regenerative Biomaterials* 2022;9:rbac019.

6. Chattopadhyay S, Raines RT. Review collagen-based biomaterials for wound healing. *Biopolymers* 2014;101:821-33.

7. Pachence JMJJoBMR. Collagen-based devices for soft tissue repair. 1998;33:35.

8. Berg RA, Silver FH, Pachence JM. Collagen matrix beads for soft tissue repair. In: US.

9. You S, Zhu Y, Li H, He F, Liu S, Yang X, Wang L, Zeng H, Dai J, Hu L. Recombinant humanized collagen remodels endometrial immune microenvironment of chronic endometritis through macrophage immunomodulation. *Regenerative Biomaterials* 2023;10.

10. Bai M, Kang N, Xu Y, Wang J, Shuai X, Liu C, Jiang Y, Du Y, Gong P, Lin H, Zhang X. The influence of tag sequence on recombinant humanized collagen (rhCol) and the evaluation of rhCol on Schwann cell behaviors. *Regenerative Biomaterials* 2023;10.

11. Gould LJ. Topical Collagen-Based Biomaterials for Chronic Wounds: Rationale and Clinical Application. *Adv Wound Care (New Rochelle)* 2016;5:19-31.

12. Wang W, Li Z, Liu J, Wang Y, Liu S, Sun M. Comparison between thermal hydrolysis and enzymatic proteolysis processes for the preparation of tilapia skin collagen hydrolysates. *Czech Journal of Food Sciences* 2013;31:1-4.

13. Huan, Yang, Haibo, Wang, Yan, Zhao, Haiyin, Wang, Hanjun, Food ZJJotSo, Agriculture. Effect of heat treatment on the enzymatic stability of grass carp skin collagen and its ability to form fibrils in vitro. 2015.

14. Han QY, Koyama T, Watabe S, Nagashima Y, Ishizaki S. Isolation and Characterization of Collagen and Collagen Peptides with Hyaluronidase Inhibition Activity Derived from the Skin of Marlin (Istiophoridae). *Molecules* 2023;28.

15. Fushimi H, Hiratsuka T, Okamura A, Ono Y, Ogura I, Nishimura I. Recombinant collagen polypeptide as a versatile bone graft biomaterial. *Communications Materials* 2020;1.

16. Gao J, Guo Z, Zhang Y, Liu Y, Xing F, Wang J, Luo X, Kong Y, Zhang G. Age-related changes in the ratio of Type I/III collagen and fibril diameter in mouse skin. *Regenerative Biomaterials* 2022;10.

17. Liu Y, Griffith M, Watsky MA, Forrester JV, Kuffova L, Grant D, Merrett K, Carlsson DJ. Properties of porcine and recombinant human collagen matrices for optically clear tissue engineering applications. *Biomacromolecules* 2006;7:1819-28.

18. Ren-Fa L, Qiang Z, Orthopaedics DO, Hospital SJCJoCR. In vitro preparation of collagen in tissue engineering and the evaluation of biocompatibility. 2005.

19. Chen Z, Fan D, Shang L. Exploring the potential of the recombinant human collagens for biomedical and clinical applications: a short review. *Biomed Mater* 2020;16:012001.

20. Qin SNJOJoC. Application of collagen in cartilage tissue engineering. 2009.

21. Fan DD, Luo Y, Mi Y, Ma XX, Shang L. Characteristics of fed-batch cultures of recombinant Escherichia coli containing human-like collagen cDNA at different specific growth rates. *Biotechnol Lett* 2005;27:865-70.

22. Chen Z, Zhang Z, Wang Z, Wu J, Wang Y, Si H, Xie X, Shang L, Fan D, Chen F. Fabricating a novel HLC-hBMP2 fusion protein for the treatment of bone defects. *J Control Release* 2021;329:270-285.

23. Cao J, Wang P, Liu Y, Zhu C, Fan D. Double crosslinked HLC-CCS hydrogel tissue engineering scaffold for skin wound healing. *Int J Biol Macromol* 2020;155:625-635.

24. Luo YE, Fan DD, Ma XX, Wang DW, Li WHJ中英. Process control for production of human-like collagen in fed-batch culture of Escherichia coli BL 21. 2005;13:4.

25. Wilkinson HN, Hardman MJ. Wound healing: cellular mechanisms and pathological outcomes. *Open Biol* 2020;10:200223.

26. Yao X, Ding J. Effects of Microstripe Geometry on Guided Cell Migration. *ACS Applied Materials & Interfaces* 2020;12:27971-27983.

27. Gu H, Li H, Wei L, Lu J, Wei Q. Collagen-based injectable and self-healing hydrogel with multifunction for regenerative repairment of infected wounds. *Regenerative Biomaterials* 2023;10.

28. Bevan D, Gherardi E, Fan TP, Edwards D, Warn R. Diverse and potent activities of HGF/SF in skin wound repair. *J Pathol* 2004;203:831-8.

29. Ohara H, Ichikawa S, Matsumoto H, Akiyama M, Fujimoto N, Kobayashi T, Tajima S. Collagen-derived dipeptide, proline-hydroxyproline, stimulates cell proliferation and hyaluronic acid synthesis in cultured human dermal fibroblasts. *J Dermatol* 2010;37:330-8.

30. Grinnell F. Fibroblasts, myofibroblasts, and wound contraction. *J Cell Biol* 1994;124:401-4.

31. Shaw TJ, Martin P. Wound repair: a showcase for cell plasticity and migration. *Curr Opin Cell Biol* 2016;42:29-37.

32. Santoro MM, Gaudino G. Cellular and molecular facets of keratinocyte reepithelization during wound healing. *Exp Cell Res* 2005;304:274-86.

33. Sunilkumar, Parameshwaraiah S, Shivakumar HG. Evaluation of topical formulations of aqueous extract of Centella asiatica on open wounds in rats. *Indian J Exp Biol* 1998;36:569-72.

34. Desmouliere A, Geinoz A, Gabbiani F, Gabbiani G. Transforming growth factor-beta 1 induces alpha-smooth muscle actin expression in granulation tissue myofibroblasts and in quiescent and growing cultured fibroblasts. *J Cell Biol* 1993;122:103-11.

35. Peters T, Sindrilaru A, Hinz B, Hinrichs R, Menke A, Al-Azzeh EA, Holzwarth K, Oreshkova T, Wang H, Kess D, Walzog B, Sulyok S, Sunderkotter C, Friedrich W, Wlaschek M, Krieg T, Scharffetter-Kochanek K. Wound-healing defect of CD18(-/-) mice due to a decrease in TGF-beta1 and myofibroblast differentiation. *EMBO J* 2005;24:3400-10.

36. Rosensteel SM, Wilson RP, White SL, Ehrlich HP. COL1A1 oligodeoxynucleotides decoy: biochemical and morphologic effects in an acute wound repair model. *Exp Mol Pathol* 2010;89:307-13.

37. Abraham, D. JJJoBC. Tumor necrosis factor alpha suppresses the induction of connective tissue growth factor by transforming growth factor-beta in normal and scleroderma fibroblasts. 2000;275:15220.

38. Abd Ghafar N, Ker-Woon C, Hui CK, Mohd Yusof YA, Wan Ngah WZ. Acacia honey accelerates in vitro corneal ulcer wound healing model. *BMC Complement Altern Med* 2016;16:259.

39. Liu CY, Lin HH, Tang MJ, Wang YK. Vimentin contributes to epithelial-mesenchymal transition cancer cell mechanics by mediating cytoskeletal organization and focal adhesion maturation. *Oncotarget* 2015;6:15966-83.

40. Fallas JA, O'Leary LE, Hartgerink JD. Synthetic collagen mimics: self-assembly of homotrimers, heterotrimers and higher order structures. *Chem Soc Rev* 2010;39:3510-27.

41. Xia Z, Das P, Shakhnovich EI, Zhou R. Collapse of unfolded proteins in a mixture of denaturants. *J Am Chem Soc* 2012;134:18266-74.

42. Abdul-Gader A, Miles AJ, Wallace BA. A reference dataset for the analyses of membrane protein secondary structures and transmembrane residues using circular dichroism spectroscopy. *Bioinformatics* 2011;27:1630-6.

43. Wei Y, Thyparambil AA, Latour RA. Quantification of the influence of protein-protein interactions on adsorbed protein structure and bioactivity. *Colloids Surf B Biointerfaces* 2013;110:363-71.

44. Gómez-Guillén MC, Turnay J, Fernández-Dı́az MD, Ulmo N, Lizarbe MA, Montero P. Structural and physical properties of gelatin extracted from different marine species: a comparative study. *Food Hydrocolloids* 2002;16:25-34.

45. Vitale N, Kisslinger A, Paladino S, Procaccini C, Matarese G, Pierantoni GM, Mancini FP, Tramontano D. Resveratrol couples apoptosis with autophagy in UVB-irradiated HaCaT cells. *PLoS One* 2013;8:e80728.

46. Yasui H, Hakozaki T, Date A, Yoshii T, Sakurai H. Real-time chemiluminescent imaging and detection of reactive oxygen species generated in the UVB-exposed human skin equivalent model. *Biochem Biophys Res Commun* 2006;347:83-8.

47. Quan T, Wang F, Shao Y, Rittie L, Xia W, Orringer JS, Voorhees JJ, Fisher GJ. Enhancing structural support of the dermal microenvironment activates fibroblasts, endothelial cells, and keratinocytes in aged human skin in vivo. *J Invest Dermatol* 2013;133:658-667.

48. Thyss R, Virolle V, Imbert V, Peyron JF, Aberdam D, Virolle T. NF-kappaB/Egr-1/Gadd45 are sequentially activated upon UVB irradiation to mediate epidermal cell death. *EMBO J* 2005;24:128-37.

49. Qian B, Li J, Guo K, Guo N, Zhong A, Yang J, Wang J, Xiao P, Sun J, Xiong L. Antioxidant biocompatible composite collagen dressing for diabetic wound healing in rat model. *Regenerative Biomaterials* 2021;8.

50. Guo X, Ma Y, Wang H, Yin H, Shi X, Chen Y, Gao G, Sun L, Wang J, Wang Y, Fan D. Status and developmental trends in recombinant collagen preparation technology. *Regenerative Biomaterials* 2023;11.

51. Ying H, Zhou J, Wang M, Su D, Ma Q, Lv G, Chen J. In situ formed collagen-hyaluronic acid hydrogel as biomimetic dressing for promoting spontaneous wound healing. *Mater Sci Eng C Mater Biol Appl* 2019;101:487-498.

52. Feng X, Zhang X, Li S, Zheng Y, Shi X, Li F, Guo S, Yang J. Preparation of aminated fish scale collagen and oxidized sodium alginate hybrid hydrogel for enhanced full-thickness wound healing. *Int J Biol Macromol* 2020;164:626-637.

53. Xing M, Fu R, Liu Y, Wang P, Ma P, Zhu C, Fan D. Human-like collagen promotes the healing of acetic acid-induced gastric ulcers in rats by regulating NOS and growth factors. *Food Funct* 2020;11:4123-4137.

54. Millauer B, Wizigmann-Voos S, Schnurch H, Martinez R, Moller NP, Risau W, Ullrich A. High affinity VEGF binding and developmental expression suggest Flk-1 as a major regulator of vasculogenesis and angiogenesis. *Cell* 1993;72:835-46.

55. Chen K, Tong C, JingeYang, Cong P, Liu Y, Shi X, Liu X, Zhang J, Zou R, Xiao K, Ni Y, Xu L, Hou M, Jin H, Liu Y. Injectable melatonin-loaded carboxymethyl chitosan (CMCS)-based hydrogel accelerates wound healing by reducing inflammation and promoting angiogenesis and collagen deposition. *Journal of Materials Science & Technology* 2021;63:236-245.

56. Chang L, Xu Y, Wu Z, Shao Y, Yu D, Yang W, Ye L, Wang X, Li B, Yin Y. Hyaluronic acid methacrylate/laponite hydrogel loaded with BMP4 and maintaining its bioactivity for scar-free wound healing. *Regenerative Biomaterials* 2023;10.

57. Chung JH, Seo JY, Choi HR, Lee MK, Youn CS, Rhie G, Cho KH, Kim KH, Park KC, Eun HC. Modulation of skin collagen metabolism in aged and photoaged human skin in vivo. *J Invest Dermatol* 2001;117:1218-24.

58. El-Domyati M, Attia S, Saleh F, Brown D, Birk DE, Gasparro F, Ahmad H, Uitto J. Intrinsic aging vs. photoaging: a comparative histopathological, immunohistochemical, and ultrastructural study of skin. *Exp Dermatol* 2002;11:398-405.

59. Liu D, Hu H, Lin Z, Chen D, Zhu Y, Hou S, Shi X. Quercetin deformable liposome: preparation and efficacy against ultraviolet B induced skin damages in vitro and in vivo. *J Photochem Photobiol B* 2013;127:8-17.

60. Sugianto M, Achadiyani A, Nugraha GI. Antioxidant Effects of Red Fruit Oil on MMP-1 Gene Expression and Malondialdehyde Levels on Skin Exposed to UVB Rays. *Molecular and Cellular Biomedical Sciences* 2019;3:100.

61. Zhang J, Hu J, Chen B, Zhao T, Gu Z. Superabsorbent poly(acrylic acid) and antioxidant poly(ester amide) hybrid hydrogel for enhanced wound healing. *Regenerative Biomaterials* 2021;8.

62. Gao J, Ning C, Wang M, Wei M, Ren Y, Li W. Structural, antioxidant activity, and stability studies of jellyfish collagen peptide–calcium chelates. *Food Chemistry: X* 2024:101706.

63. Li Y, Jiang B, Zhang T, Mu W, Liu J. Antioxidant and free radical-scavenging activities of chickpea protein hydrolysate (CPH). *Food Chemistry* 2008;106:444-450.

64. Zhang J, Li M, Zhang G, Tian Y, Kong F, Xiong S, Zhao S, Jia D, Manyande A, Du H. Identification of novel antioxidant peptides from snakehead (Channa argus) soup generated during gastrointestinal digestion and insights into the anti-oxidation mechanisms. *Food Chemistry* 2021;337:127921.

65. Li C, Fu Y, Dai H, Wang Q, Gao R, Zhang Y. Recent progress in preventive effect of collagen peptides on photoaging skin and action mechanism. *Food Science and Human Wellness* 2022;11:218-229.

66. Herawati E, Akhsanitaqwim Y, Agnesia P, Listyawati S, Pangastuti A, Ratriyanto A. In Vitro Antioxidant and Antiaging Activities of Collagen and Its Hydrolysate from Mackerel Scad Skin (Decapterus macarellus). *Marine Drugs,* 10.3390/md20080516.

67. Zhao X, Zhang X, Liu D. Collagen peptides and the related synthetic peptides: A review on improving skin health. *Journal of Functional Foods* 2021;86:104680.

68. Guoying Y, Pengli C, Kun G, Yuejun W, Mi S, Chunbo W. Effects of polypeptide fromChlamys farreri on amino acid content in guinea pig skin irradiated by chronic ultraviolet A and B. *Chinese Journal of Oceanology and Limnology* 2004;22:440-442.

69. Wang J, Qiu H, Xu Y, Gao Y, Tan P, Zhao R, Liu Z, Tang Y, Zhu X, Bao C, Wang H, Lin H, Zhang X. The biological effect of recombinant humanized collagen on damaged skin induced by UV-photoaging: An in vivo study. *Bioact Mater* 2022;11:154-165.

70. Kochanek-K S, Brenneisen P, Wenk J, Herrmann G, Ma W, Kuhr L, Meewes C, Wlaschek MJEG. Photoaging of the skin from phenotype to mechanisms. 2000:35.

71. Park HM, Moon E, Kim AJ, Kim MH, Lee S, Lee JB, Park YK, Jung HS, Kim YB, Kim SY. Extract of Punica granatum inhibits skin photoaging induced by UVB irradiation. *Int J Dermatol* 2010;49:276-82.

72. Hwang E, Lee DG, Park SH, Oh MS, Kim SY. Coriander leaf extract exerts antioxidant activity and protects against UVB-induced photoaging of skin by regulation of procollagen type I and MMP-1 expression. *J Med Food* 2014;17:985-95.

73. Kang TH, Park HM, Kim YB, Kim H, Kim N, Do JH, Kang C, Cho Y, Kim SY. Effects of red ginseng extract on UVB irradiation-induced skin aging in hairless mice. *J Ethnopharmacol* 2009;123:446-51.

74. Hu Z, Yang P, Zhou C, Li S, Hong P. Marine Collagen Peptides from the Skin of Nile Tilapia (Oreochromis niloticus): Characterization and Wound Healing Evaluation. *Mar Drugs* 2017;15.

75. Udhayakumar S, Shankar KG, Sowndarya S, Rose C. Novel fibrous collagen-based cream accelerates fibroblast growth for wound healing applications: in vitro and in vivo evaluation. *Biomaterials Science* 2017;5:1868-1883.

76. Felician FF, Yu R-H, Li M-Z, Li C-J, Chen H-Q, Jiang Y, Tang T, Qi W-Y, Xu H-M. The wound healing potential of collagen peptides derived from the jellyfish Rhopilema esculentum. *Chinese Journal of Traumatology* 2019;22:12-20.

77. Xiao M, Gao L, Chandrasekaran AR, Zhao J, Tang Q, Qu Z, Wang F, Li L, Yang Y, Zhang X, Wan Y, Pei H. Bio-functional G-molecular hydrogels for accelerated wound healing. *Materials Science and Engineering: C* 2019;105:110067.

78. Wang J, Qiu H, Xu Y, Gao Y, Tan P, Zhao R, Liu Z, Tang Y, Zhu X, Bao C, Wang H, Lin H, Zhang X. The biological effect of recombinant humanized collagen on damaged skin induced by UV-photoaging: An in vivo study. *Bioactive Materials* 2022;11:154-165.

79. Ni H, Liu C, Kong L, Zhai L, Chen J, Liu Q, Chen Z, Wu M, Chen J, Guo Y, Bai W, Zhang D, Xia K, Huang G, Pan S, Liao B, Ma K, Zhang L-K, Cheng J, Guan Y-Q. Preparation of injectable porcine skin-derived collagen and its application in delaying skin aging by promoting the adhesion and chemotaxis of skin fibroblasts. *International Journal of Biological Macromolecules* 2023;253:126718.

80. Fu C, Shi S, Tian J, Gu H, Yao L, Xiao J. Non-denatured yak type I collagen accelerates sunburned skin healing by stimulating and replenishing dermal collagen. *Biotechnology Reports* 2023;37:e00778.

81. Huang J-j, Li H-l, Xiong G-q, Cai J, Liao T, Zu X-y. Extraction, identification and anti-photoaging activity evaluation of collagen peptides from silver carp (Hypophthalmichthys molitrix) skin. *LWT* 2023;173:114384.

82. Davison-Kotler E, Marshall WS, García-Gareta E. Sources of Collagen for Biomaterials in Skin Wound Healing. *Bioengineering (Basel)* 2019;6.

83. Maher M, Castilho M, Yue Z, Glattauer V, Hughes TC, Ramshaw JAM, Wallace GG. Shaping collagen for engineering hard tissues: Towards a printomics approach. *Acta Biomaterialia* 2021;131:41-61.

84. Majkowska I, Shitomi Y, Ito N, Gray NS, Itoh Y. Discoidin domain receptor 2 mediates collagen-induced activation of membrane-type 1 matrix metalloproteinase in human fibroblasts. *Journal of Biological Chemistry* 2017;292:6633-6643.

85. Olaso E, Ikeda K, Eng FJ, Xu L, Wang LH, Lin HC, Friedman SL. DDR2 receptor promotes MMP-2-mediated proliferation and invasion by hepatic stellate cells. *J Clin Invest* 2001;108:1369-78.
