# Supplementary material for: Recombinant collagen for the repair of skin wounds and photo-aging damage
Source: Regen Biomater. 2024 Sep 2;11:rbae108. doi: 10.1093/rb/rbae108 (PMC11422187; doi:10.1093/rb/rbae108)
Supplement: rbae108_Supplementary_Data [file rbae108_supplementary_data.zip › Supplementary information.docx]

Supplementary information

**Recombinant collagen for the repair of skin wounds and photo-aging damage**

Taishan Liu^#,1,2,3^, Jiayun Hao ^#,1,2,3^，Huan Lei^#,1,2,3^，Yanru Chen^1,2,3^，Lin Liu^4^，Liping Jia^4^，Juan Gu^5^，Huaping Kang^5^，Jingjing Shi^5^，Jing He^4^，Yangbin Song^5^，Yuqi Tang^5^，Daidi Fan^*,1,2,3^

^1^Shaanxi Key Laboratory of Degradable Biomedical Materials, School of Chemical Engineering, Northwest University, Taibai North Road 229, Xi’an 710069, China.

^2^Shaanxi R&D Center of Biomaterials and Fermentation Engineering, School of Chemical Engineering, Northwest University, Xi’an 710069, China.

^3^Biotech & Biomed Research Institute, Northwest University, Xi’an 710069, China.

^4^Xi’an Giant Biotechnology Co.LTD, Shanglinyuan Road, Xi’an 710100, China.

^5^Shaanxi Giant Biotechnology Co.LTD, Jinye Road, Xi'an, 710076, China.

^#^These authors contributed equally to this work

^*^Corresponding Author

Daidi Fan (Corresponding Author, ORCID: 0000-0001-9798-1674)

E-mail: [fandaidi@nwu.edu.cn](mailto:fandaidi@nwu.edu.cn)


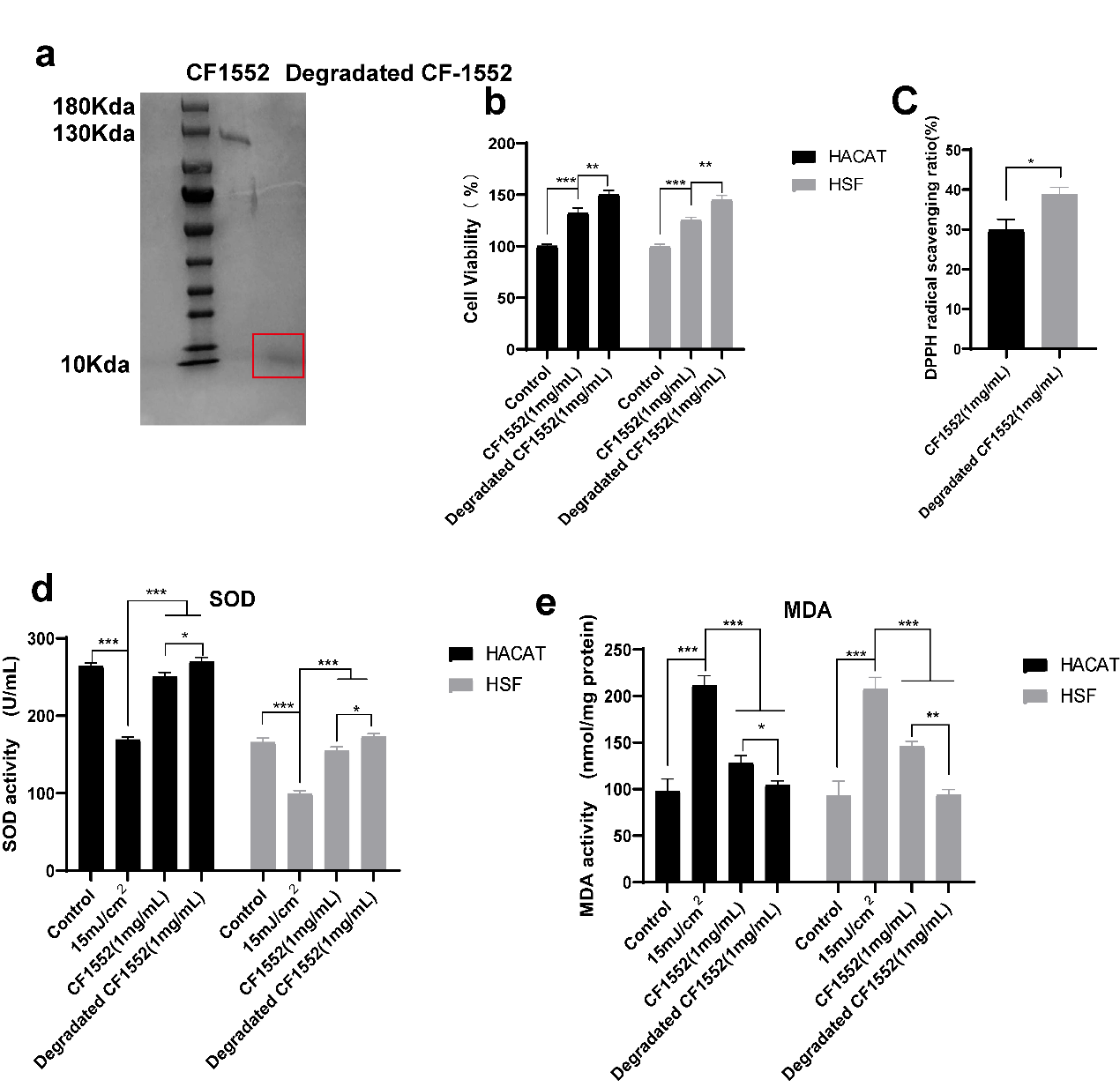


Fig.S1 Preliminary study on the biological activity of CF-1552（I）degradation products, a) SDS-page electrophoresis images of CF-1552（I）(left) and its hydrolysate (right),b）Cell viability (HACAT and HSF) with 1mg/mL CF1552（I）and its degradation products(1mg/mL),n=3, (*p < 0.05, **p < 0.01, ***p < 0.001),c) DPPH scavenging rate of CF-1552（I）(1mg/mL) and its hydrolysate (1mg/mL) n=3, (*p < 0.05, **p < 0.01, ***p < 0.001),d) Superoxide dismutase (SOD) activity of CF-1552（I）(1mg/mL) and its degradation products(1mg/mL), n=3, (*p < 0.05, **p < 0.01, ***p < 0.001),e) Malonaldehyde (MDA) activity of CF-1552（I）(1mg/mL) and its degradation products(1mg/mL), n=3, (*p < 0.05, **p < 0.01, ***p < 0.001). All the results from 3 independent experiments are presented as the mean ± SD.

Table S1 Comparison of skin repair effects of different sources of collagen

| Serial No. | Sample form | Types of collagen | Availability of other functional materials | Functions of recombinant collagen and animal collagen in this system, content | Comparisons in cellular experiments | Wound repair time/efficiency | Comparison of data on wound repair mechanisms | Reference |
| --- | --- | --- | --- | --- | --- | --- | --- | --- |
| 1 | collagen solution | Collagen from fish skin | none | Wound healing evaluation（ 6.25 to 50.0 mg/mL） | In-vitro studies revealed thatfish skin collagen shows significant wound closure at concentration 50.0 mg/mL, after treatment for 12, 18 and 24 h | The in vivo study showed that the wound healing rate was higher in the fish skin collagen group than in the model control and positive control groups, with (38.8% ± 22.8%), (8.7% ± 17.2%) and (19.5% ± 35.0%), respectively. | antiinflammation, cell proliferation↑ | [1] |
| 2 | collagen-based cream | Bovine Col type-I | chitosan | Wound healing evaluation | In vitro studies have shown that bovine collagen exhibits 95% cell survival under fluorescence spectroscopy compared to controls | In vivo studies have shown that wounds treated with bovine collagen can be fully re-epithelialized in only 16 days compared to controls, which took nearly 19-21 days. | none | [2] |
| 3 | collagen solution | collagen peptides | none | examining wound contraction, re-epithelialization, tissue regeneration  (0.9g/kg bodyweight） | Wound scratch assay showed that there were significant effects on the scratch closure on cells treated with collagen peptides at a concentration of 6.25μg/mL for 48h as compared to the vehicle treated cells | Wound area contraction after intragastric injection of collagen peptide (0.9 g/kg bw) in mice on day 8. | Increased TGF-β1 and b-FGF | [3] |
| 4 | hydrogel | Recombinant Human Collagen  type III | guanosine | To speed up the wound healing process（5mg/mL） | The wound closure rate of scratch wounds in the hydrogel group was 50%, showing a significant difference between the experimental and control groups. | Guanosine quartet recombinant human Col hydrogel showed a 60% increment in wound closure on the 3rd day, 78% closure on the 5th day, and on day 7th, wound closure reached about 91%. | anti-inflammation  cell proliferation↑ | [4] |
| 5 | collagen solution | Recombinant Human Collagen  type III | none | skin damage repair.（0.8 mg/mL，2mg/mL,3.2mg/mL） | none | In the experimental group with medium concentrations of rhCol III, collagen content increased by 3.4%, 12.4%, and 3.0% at 3, 5, and 8 weeks postoperatively, respectively | antioxidant↑  collagen expression↑  MMP3↓,SOD↑  MDA↓，HYP↑ | [5] |
| 6 | collagen solution | porcine skin-derived collagen | none | Collagen promotes adhesion and chemotaxis of skin fibroblasts（0,1 mg/mL,3 mg/mL） | promote the adhesion and chemotaxis of HSF cells and provide a good extracellular environment for cell growth | Porcine skin collagen could effectively alleviate cell senescence *in vitro*. In old rats, the thickness of the epidermis decreased and the number of dermal fibroblasts increased to restore cell morphology. | Antioxidant↑  anti-aging | [6] |
| 7 | collagen solution | Non-denatured yak type I collagen | none | accelerates sunburned skin healing (0.01 ~ 1.0 mg/mL) | The relative proliferation rate of human skin fibroblasts (HFF-1) in 0.1 mg/mL collagen solution gradually increased to 120% on day 3 and 150% on day 5, indicating that YCI significantly promoted the proliferation of HFF-1. | has a significant accelerated healing effect on sunburned skin on the fourth day. | Antioxidant↑，collagen expression↑  MDA↓ | [7] |
| 8 | collagen solution | collagen peptides from silver carp skin | none | Effect on proliferation and migration of L929 cells | Cell viability values ranged from 94.8 -107.2% (0.05-1 mg/mL), with the highest cell scratch wound closure rate (64.6%) | none | none | [8] |
| This study | collagen solution | COLI&CF-1552 | none | Promote cell proliferation migration antioxidant (0, 0.2, 0.4, 0.8 and 1 mg/mL) | For HACAT cells, the proliferation rates of 1 mg/mL ColI and CF-1552 after 24 hours were 127.35% and 132.2%; the cell scratch healing rates were 37.5% and 29.5%, respectively; for HSF cells, the proliferation rates of 1 mg/mL ColI and CF-1552 after 24 hours were 123.22% and 125.37%; the cell scratch healing rates were 42.5% and 45.5%, respectively. 125.37%; the cell scratch healing rates were 42.5% and 45.5%, respectively. | Both ColI and CF1552 cause complete healing of total cortical wounds at 13 days and promote UV aging repair by down-regulating MMP1 and up-regulating HYP | Antioxidant↑  collagen expression↑  MDA↓ |  |

# Reference

1. Hu Z, Yang P, Zhou C, Li S, Hong P. Marine Collagen Peptides from the Skin of Nile Tilapia (Oreochromis niloticus): Characterization and Wound Healing Evaluation. *Mar Drugs* 2017;15.

2. Udhayakumar S, Shankar KG, Sowndarya S, Rose C. Novel fibrous collagen-based cream accelerates fibroblast growth for wound healing applications: in vitro and in vivo evaluation. *Biomaterials Science* 2017;5:1868-1883.

3. Felician FF, Yu R-H, Li M-Z, Li C-J, Chen H-Q, Jiang Y, Tang T, Qi W-Y, Xu H-M. The wound healing potential of collagen peptides derived from the jellyfish Rhopilema esculentum. *Chinese Journal of Traumatology* 2019;22:12-20.

4. Xiao M, Gao L, Chandrasekaran AR, Zhao J, Tang Q, Qu Z, Wang F, Li L, Yang Y, Zhang X, Wan Y, Pei H. Bio-functional G-molecular hydrogels for accelerated wound healing. *Materials Science and Engineering: C* 2019;105:110067.

5. Wang J, Qiu H, Xu Y, Gao Y, Tan P, Zhao R, Liu Z, Tang Y, Zhu X, Bao C, Wang H, Lin H, Zhang X. The biological effect of recombinant humanized collagen on damaged skin induced by UV-photoaging: An in vivo study. *Bioactive Materials* 2022;11:154-165.

6. Ni H, Liu C, Kong L, Zhai L, Chen J, Liu Q, Chen Z, Wu M, Chen J, Guo Y, Bai W, Zhang D, Xia K, Huang G, Pan S, Liao B, Ma K, Zhang L-K, Cheng J, Guan Y-Q. Preparation of injectable porcine skin-derived collagen and its application in delaying skin aging by promoting the adhesion and chemotaxis of skin fibroblasts. *International Journal of Biological Macromolecules* 2023;253:126718.

7. Fu C, Shi S, Tian J, Gu H, Yao L, Xiao J. Non-denatured yak type I collagen accelerates sunburned skin healing by stimulating and replenishing dermal collagen. *Biotechnology Reports* 2023;37:e00778.

8. Huang J-j, Li H-l, Xiong G-q, Cai J, Liao T, Zu X-y. Extraction, identification and anti-photoaging activity evaluation of collagen peptides from silver carp (Hypophthalmichthys molitrix) skin. *LWT* 2023;173:114384.
